# Lipopolysaccharide-induced interferon response networks at birth are predictive of severe viral lower respiratory infections in the first year of life

**DOI:** 10.3389/fimmu.2022.876654

**Published:** 2022-08-05

**Authors:** James F. Read, Michael Serralha, Danny Mok, Barbara J. Holt, Mark Cruickshank, Yuliya V. Karpievitch, David I. Broadhurst, Peter D. Sly, Deborah H. Strickland, Stacey N. Reinke, Patrick G. Holt, Anthony Bosco

**Affiliations:** ^1^ Telethon Kids Institute, The University of Western Australia, Perth, WA, Australia; ^2^ School of Medicine, The University of Western Australia, Nedlands, WA, Australia; ^3^ School of Biomedical Sciences, The University of Western Australia, Nedlands, WA, Australia; ^4^ Centre for Integrative Metabolomics & Computational Biology, School of Science, Edith Cowan University, Joondalup, WA, Australia; ^5^ Child Health Research Centre, The University of Queensland, Brisbane, QLD, Australia; ^6^ The University of Western Australia Centre for Child Health Research, The University of Western Australia, Nedlands, WA, Australia; ^7^ Asthma and Airway Disease Research Center, University of Arizona, Tucson, AZ, United States; ^8^ Department of Immunobiology, The University of Arizona College of Medicine, Tucson, AZ, United States

**Keywords:** innate immunity, respiratory infection, interferon, lipopolysaccharide (LPS), multi-omics, systems biology, pathogen recognition receptor (PRR)

## Abstract

Appropriate innate immune function is essential to limit pathogenesis and severity of severe lower respiratory infections (sLRI) during infancy, a leading cause of hospitalization and risk factor for subsequent asthma in this age group. Employing a systems biology approach to analysis of multi-omic profiles generated from a high-risk cohort (n=50), we found that the intensity of activation of an LPS-induced interferon gene network at birth was predictive of sLRI risk in infancy (AUC=0.724). Connectivity patterns within this network were stronger among susceptible individuals, and a systems biology approach identified IRF1 as a putative master regulator of this response. These findings were specific to the LPS-induced interferon response and were not observed following activation of viral nucleic acid sensing pathways. Comparison of responses at birth versus age 5 demonstrated that LPS-induced interferon responses but not responses triggered by viral nucleic acid sensing pathways may be subject to strong developmental regulation. These data suggest that the risk of sLRI in early life is in part already determined at birth, and additionally that the developmental status of LPS-induced interferon responses may be a key determinant of susceptibility. Our findings provide a rationale for the identification of at-risk infants for early intervention aimed at sLRI prevention and identifies targets which may be relevant for drug development.

## Introduction

Severe lower respiratory tract infections (sLRIs) are a leading cause of emergency room presentations by infants and children (1, 2), and are a major risk factor for the development of asthma and wheeze (3–6). Rhinovirus (RV) and Respiratory Syncytial Virus (RSV) are the most important triggers of early life respiratory infections and asthma development (7–9). Several studies have found that associations between sLRI and asthma are strongest in children with RV-related wheezing and early aeroallergen sensitization (5, 6, 10, 11), although a causal relationship remains to be established (7). However, RV can routinely be detected in asthmatic children in the absence of asthmatic symptoms, suggesting that RV may be necessary but not sufficient to drive the pathogenesis of asthma (12). In this regard it has been demonstrated that bacterial pathogens, including *Moraxell*a, *Streptococcus*, and *Haemophilus* species, are important triggers of wheezy episodes in young children (13, 14), and may also contribute towards asthma inception (15, 16). Furthermore, the presence of pathogenic bacteria in the nasopharynx during acute respiratory viral infections may increase the likelihood of infection spread to the lower airways, amplifying ensuing inflammatory symptoms and increasing risk of subsequent asthma development (17, 18), although much remains unknown regarding virus-bacteria interactions in the airways and asthma development (19, 20). Conversely, exposure to microbes and their products during early childhood has also been shown to protect against asthma, perhaps most elegantly illustrated through the “farm effect” (21, 22). The underlying immunological mechanisms that determine why some individuals are more susceptible to sLRIs in early life, and subsequent asthma, are not well understood. Innate immune function in the immediate postnatal period, which experiences drastic developmental changes (8, 23–25), provides a logical link between early life microbial exposure and infection susceptibility. This has prompted investigation of molecular events downstream of pathogen recognition receptor (PRR) activation, such as Toll-like receptors (TLRs), in blood collected at birth and in early life (26–31). For example, reduced type 1/3 interferon response capacity following stimulation with Polyinosinic-polycytidylic acid (Poly(I:C)) – a potent activator of TLR3 – in cord blood cells is associated with increased risk for febrile LRIs and early childhood wheeze (31). Moreover, enhanced production of the proinflammatory cytokine IL-1β following bacterial lipopolysaccharide (LPS) activation of cord blood was observed in individuals at risk of childhood-onset asthma, in association with increased *SMAD3* methylation and maternal asthma status (30). These examples reveal that aspects of innate immunity which may confer risk for sLRIs and subsequent asthma are already detectable at birth. The aim of the present study was to determine if innate immune response profiles induced by bacterial LPS or viral nucleic acid sensing pathways (Poly(I:C) and Imiquimod) at birth could predict sLRI in the first year of life. The rationale for selecting these pathways is that previous studies have highlighted the role of bacteria, respiratory viral infections, and innate immune responses to the selected TLR agonists in asthma risk (7, 9, 15, 30–33).

## Materials and methods

### Study population

Subjects were a subset of 50 individuals from the Childhood Asthma Study, a 10 year prospective birth cohort enrolled prenatally for high risk of asthma development, as described previously (5, 31, 34–36). Of the 60 subjects with at least one CBMC aliquot remaining from the cohort, nine were excluded due to insufficient information (withdrawn before the 1 year follow-up) and a further subject was excluded for insufficient sample volume. Acute respiratory infections were considered sLRIs if wheeze and/or fever was present in addition to chest rattle, as this definition has previously been linked to persistent wheeze and asthma in this cohort (5, 35) (**Supplementary Methods**). Respiratory viral infection histories were determined from detailed assessment and nasopharyngeal aspirates (RT-PCR) collected during home visits within 48 hours of symptom development (5, 34). Ethics was approved by The University of Western Australia (reference RA/4/1/7560), and fully informed parental consent was obtained for all subjects.

### Immunophenotyping

Approximately 1x10^6^ CBMCs from each sample were immunophenotyped with a panel of 11 monoclonal antibodies to measure CD3, CD4, CD11c, CD14, CD19, CD25, CD123, CD127, FcϵRIα, FoxP3, and HLA-DR. Individual cells were acquired using the LSRFortessa platform with FACSDiva software (BD Biosciences) following quality control measures. FlowJo (v10.5) software and R were used for pre-processing and analysis (**Supplementary Methods**).

### *In vitro* cell culture

Samples were assigned randomized blocks and cultured sequentially by the same personnel using consistent reagent/stimuli stocks. Cord blood erythrocytes were immunomagnetically depleted (EasySep kit, StemCell, Cat no. 18170) and each sample was cultured in RPMI 1640 (Gibco, Cat No. 11875119) supplemented with 5% AB serum (Sigma-Aldrich, Cat. No. H3667) for 18 hours (37°C, 5% CO_2_) with 1ng/ml LPS (Enzo Biochem, Cat No. ALX-581-007-L001; derived from E. *coli*, serotype R515), 5μl/ml Imiquimod (*In vivo* Gen, Cat. Code: tlrl-imq) or 50μl/ml Poly(I:C) (*In vivo* Gen, Cat. Code: tlrl-pic), alongside matched unstimulated controls. Poly(I:C), bacterial LPS, and Imiquimod were selected as they are activators of TLRs 3, 4, and 7, respectively, thereby triggering innate immune responses to LPS and viral nucleic acid sensing pathways. However, it is noteworthy that Poly(I:C), a synthetic analogue of double-stranded RNA, can also activate the viral-related PRRs RIG-I and MDA5 (37). Aliquots of culture supernatant were immediately snap frozen in liquid nitrogen for metabolomic profiling or stored at -20°C for cytokine quantification. Cell pellets were stored in TRIzol reagent (Invitrogen, Cat No. 15596026) at -20°C for RNA extraction.

### Data generation

Detailed information on sample and data processing and quality control are available in the **Supplementary Methods**.

RNA-Seq: RNA was purified with RNeasy MinElute Kits (Qiagen, Cat No. 74204) and libraries were prepared with NEBNext Ultra II Kits (NE BioLabs) for sequencing on the NovaSeq 6000 (Illumina) platform. Standard methods were applied for pre-processing, alignment (GRCh38), and transcript quantification. RNA-Seq data is available from the NCBI Gene Expression Omnibus repository (38) (accession number GSE184383).

Cytokines: The concentrations of 48 cytokines (Bio-plex Pro, BioRad, Cat. No: 12007283) were simultaneously quantified with the Luminex 200 system (Luminex). Nine cytokines were outside the limit of detection in >20% of stimulated samples and were removed. Raw and processed data are provided in **Data S1**.

Metabolites: Untargeted metabolomic data was generated with liquid chromatography (HILIC and C18 modes) separation coupled to a Q Exactive Orbitrap mass spectrometer (Thermo Fisher Scientific) in electrospray positive ionisation mode (LC-MS/MS). QC samples were interspersed throughout the run order to assess and correct variability. Metabolites were filtered according to stringent statistical and annotation thresholds (39). Raw and processed data are provided in **Data S2**.

### Transcriptomic analysis

Differentially expressed genes (DEG) were identified [EdgeR (40)] using an absolute log_2_ fold change >1.5 and an adjusted P value < 0.01 (Benjamini-Hochberg adjusted False Discovery Rate). Moderated t-statistics were calculated with limma/voom (41). Pathways analysis of upregulated/downregulated genes was performed with InnateDB (42). Cellular composition was estimated from post-culture gene expression with CIBERSORTx (43) with single cell RNA-seq cord blood reference profiles curated from the Human Cell Atlas (44). Gene expression data was partitioned into response context-specific modules with WGCNA (45). Modules were annotated by employing a consensus approach derived from Gene Ontology (GOenrichmentAnalysis), ReactomePA (46), and clusterProfiler (47) R packages, and InnateDB (42). Additionally, we employed the blood transcriptional module repertoire from BloodGen3Module to confirm that the principal modules of interest related to innate immune function we identified following WGCNA analysis (interferon and proinflammatory modules) were captured with an independent method (48). Separate response networks were created for each condition and included matched unstimulated controls, as detailed in the **Supplementary Methods**.

### Master regulator analysis

To identify transcription factors that act as master regulators of gene expression profiles, a gene regulator network was reverse engineered with ARACNe (49) and transcription factor activity was inferred with VIPER (50) (detailed in **Supplementary Methods**). Significant TFs (p<0.05) were considered drivers of the responses if they had known binding motifs in the region of regulon target genes (500bp upstream and 100bp downstream) determined by RcisTarget (51). Normalised expression scores (NES) outputted from VIPER were retained for downstream analysis.

### Machine learning

Gene expression data was randomly assigned into training and validation sets and filtered to only the respective module genes for each analysis. The random assignment was 50% training/50% validation. The RandomForest R package was used for random forest model construction, and the number of decision trees (ntree) and candidate variables (mtry) were optimized according to the out-of-bag error rate (**Supplementary Methods**). Model construction and classification was repeated thousands of times after randomly re-assigning samples into train/validation groups (retaining the original optimized parameters) and this was repeated with 60/40 and 70/30 splits (**Supplementary Methods**). This step was included to ensure the results were robust with respect to the randomized train/test group assignment. The same random assignment with respect to individual subjects was applied for LPS/CTRL, Poly(I:C)/CTRL, and Imiquimod/CTRL models. For random forest models used to predict infection status in independent cohorts of infant/childhood infection, CAS cohort data was filtered to respective module genes and used as the training set, and the external gene expression data was used for validation (filtered to identical input genes).

### Multi-omic data integration

A DIABLO (52) model was constructed for supervised multi-omic data integration, which generalizes Partial Least Squares analysis to maximize co-expression between matched datasets. All datasets (except immunophenotyping) for LPS-stimulated CBMC samples were baseline adjusted prior to analysis, and gene expression data was filtered to significantly variable genes (n=6344) to reduce noise. The number of components and feature selection parameters were tuned with 5x cross-validation (**Supplementary Methods**).

## Results

### Study population

**Figure 1:**
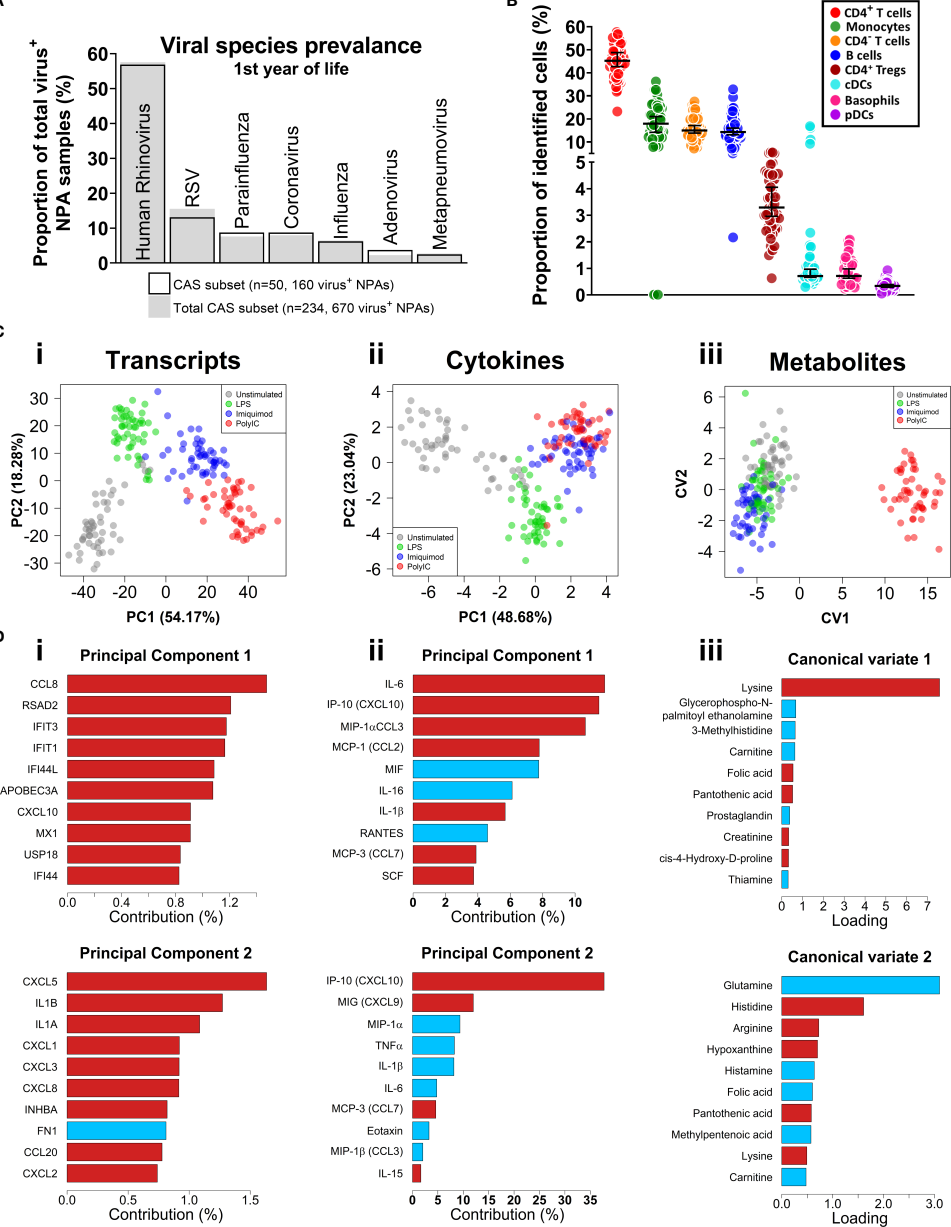
Respiratory virus prevalence, baseline CBMC cell proportions, multi-omic data set overview. **(A)** Bar plot of viral agents detected from infectious NPAs taken during year one (proportion of total virus^+^ NPAs). Bars denote study subset (n=50), grey fill represents individuals who recorded an sLRI in year 1 from the total cohort (n=234). **(B)** Immunophenotyping of baseline CBMC samples. Y-axis shows cell type as a proportion of identified cells. Scatterplots shows median and 95% CI. **(C)** Multi-level dimensionality reduction for gene expression (PCA), cytokine (PCA), and metabolite (cross validated CVA) datasets. Axes show coordinates of the first (x-axis) and second (y-axis) components/variates. **(D)** Horizontal bar plots showing top contributing features for the first **(i-iii)** and second **(iv-vi)** principal components or canonical variates for the corresponding plots in **(C)**, above. X-axis shows absolute contribution (%)/loading; red/blue indicates positive/negative relative contribution.

The study population consisted of a subset of 50 children within the Childhood Asthma Study (CAS) cohort (5, 34–36, 53). 23 subjects (46%) experienced at least one wheezy and/or febrile sLRI in their first year (infancy) and this was the primary outcome of interest (**Table 1**). These individuals typically experienced 1 or 2 sLRIs in the first year of life, and a similar number recorded both wheezy and febrile (8/23, 34.8%), wheezy only (7/23, 30.4%), and febrile only (8/23, 34.8%) sLRIs (**Table S1** and **Figure S1A**). No difference was observed with respect to sex, gestational weeks, birth weight, skin prick test positivity to common aeroallergens, and URIs in infancy for the primary outcome (**Table S1**). Overall, this subset was found to be representative of the total CAS cohort (n=263) with respect to key clinical characteristics (**Table 1**). RV was the most frequent viral agent identified from the first year of life in this subset (present in 56.9% of infectious nasopharyngeal samples) followed by RSV (13.125%), and this was representative of the total cohort (**Figures 1**, **S1**).

**Table 1:** Characteristics and representativeness of the study subset.

	CAS subset (n=50)	Total CAS cohort (n=263)	OR (95% CI)	P value
**Sex (female)**	24/50 (48%)	115/251 (45.82%)	0.92 (0.48-1.77)	0.88
**Gestation (weeks; mean [range])**	39.14 [36-41]	39.03 [34-41]	NA	0.89
**Birth weight (grams; mean [range])**	3496.52 [2755-4415]	3406.17 [2085-5110]	NA	0.27
**SPT^+^ at 0.5, 2, or 5 years**	24/50 (48%)	118/198 (59.6%)	1.59 (0.82-3.13)	0.15
**URI in first year**	47/50 (94%)	215/235 (91.49%)	0.69 (0.13-2.46)	0.78
**LRI in first year**	39/50 (78%)	160/235 (68.08%)	0.6 (0.26-1.28)	0.18
**sLRI in first year**	23/50 (46%)	101/235 (42.98%)	0.88 (0.46-1.72)	0.75
**Current wheeze at 5 years**	14/43 (32.56%)	56/198 (28.28%)	0.82 (0.38-1.8)	0.58
**Asthma at 5 years**	9/34 (26.47%)	37/198 (18.69%)	0.64 (0.26-1.69)	0.35

CAS, Childhood Asthma Study; OR, Odds Ratio; CI, Confidence Interval; URI, Upper respiratory Infection (viral); (s)LRI, (severe) Lower respiratory Infection (viral); SPT, Skin Prick Test. SPT positivity was determined from a panel of seven common allergens (house dust mite, cat dander, ryegrass, Alternaria, Aspergillus, cow’s milk, and egg white), along with positive (Histamine) and negative (saline) controls at 6 months, 2 years, and 5 years (**Supplementary Methods**). sLRIy1 represents the primary outcome (sLRI incidence in the first year of life). For categorical variables, odds ratios, 95% CIs and accompanying P values were determined by Fishers Exact test. For continuous variables, P values were determined by Mann-Whitney U test. Variation in participant number relates to data availability (see *Methods*).

### Baseline flow cytometry

We applied an 11-colour flow cytometry panel to baseline cord blood mononuclear cell (CBMC) samples to assess cellular composition. Lymphocytes (T and B cells) composed the majority of cell types identified among CBMC (**Figures 1B** and **S1B**). CD14^+^ monocytes and conventional dendritic cells (cDC) were identified among the myeloid compartment, and smaller proportions of plasmacytoid DCs (pDC) and basophils were also identified. The was no difference in baseline cellular composition with respect to sLRI in the first year of life (**Figure S1C**).

### Multi-omic profiling of innate immune responses in CBMC

CBMC from all 50 subjects were cultured for 18 hours with LPS, or Imiquimod, or Poly(I:C) to trigger innate immune responses, along with unstimulated controls. This timepoint was selected to capture signalling cascades downstream of the immediate and secondary response programs (54–56). Gene expression was profiled from cell pellets (RNA-Seq) and supernatants were used to profile cytokines (multiplex assay) and metabolites (LC-MS/MS). Matching PBMC samples collected at age 5 were available for a subset of the subjects (n = 27), and these were cultured in parallel under the same conditions. Following data pre-processing and filtering, 17,363 transcripts, 39 cytokines, and 47 metabolites were available for analysis (see Methods). We applied unsupervised Principal Component Analysis (PCA; transcripts, cytokines) and supervised Canonical Variate Analysis (CVA; metabolites) dimensionality reduction for exploratory data analysis. The samples from each biological layer clustered by stimuli as expected (**Figure 1C**). For transcripts and cytokines, the first two principal components captured interferon (IFN) and proinflammatory features (e.g., *CXCL10*/IP-10, IL-1β, IL-6) (**Figure 1D**). Poly(I:C)-stimulated cord blood sample clustering by metabolites was driven by lysine on the first canonical variate, and other amino acids (e.g., glutamine, histidine) were identified for the second and third canonical variates (**Figures 1C, D**, **S1D,E**).

### IFN and proinflammatory gene expression programs are upregulated in CBMC responses

**Figure 2:**
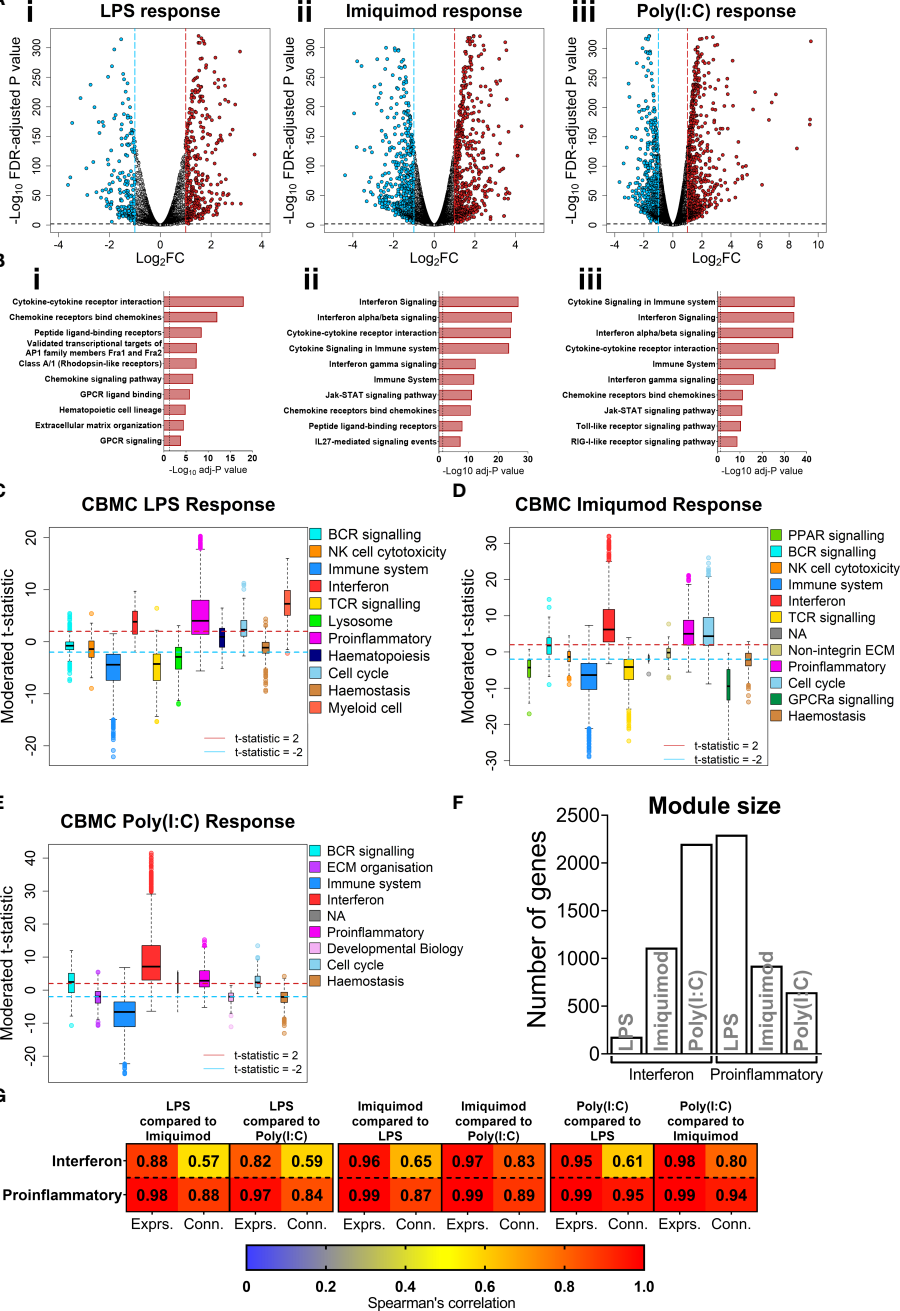
IFN and proinflammatory gene expression following TLRs 3/4/7 activation in CBMC. **(A)** Volcano plot showing significantly upregulated (red) and downregulated (blue) genes compared to matched unstimulated samples for the LPS (i), Imiquimod (ii), and Poly(I:C) (iii) responses, respectively. Plots show the Log_2_ fold-change (x-axis) and FDR-adjusted p value (-Log_10_ transformed). Blue and red dashed lines indicate a Log_2_FC of -1 and 1, respectively; black dashed line denotes a -Log_10_ FDR-adjusted p value of 2. **(B)** Top 10 overrepresented pathways from significantly upregulated genes of the CBMC LPS (i), Imiquimod (ii), and Poly(I:C) (iii) responses. X-axis shows the FDR corrected p value (-Log_10_ transformed); black dashed line indicated corrected p ≈ 0.05. **(C–E)** Modules identified from network analysis (WGCNA) of the LPS, Imiquimod, and Poly(I:C) responses, respectively. Modules are plotted by moderated t-statistics (y-axis) and show the median, 25^th^ and 75^th^ quartiles ±1.5xIQR and outliers. Modules with medians above the red line (moderated t-statistic = 2) are considered significantly upregulated and those below the blue line (-2) are considered significantly downregulated. **(F)** Bar plot of the number of genes in the interferon and proinflammatory modules for the respective responses. **(G)** Heatmap showing Spearman’s correlation values of ranked expression and ranked connectivity between CBMC response module genes. Expression of member genes from the IFN and proinflammatory modules of each response were correlated against the expression of the same genes from the other responses. The p value associated with all correlations was < 0.01.

We focused on the transcriptomics data to further investigate cord blood responses to LPS, Poly(I:C), and Imiquimod treatment as these data provide genome-wide coverage. Employing differential expression analysis, we identified 641 differently expressed genes (DEGs) for the cord blood LPS response (Log_2_-fold change > 1, FDR adjusted-P value < 0.01), and greater than 1000 DEGs for the imiquimod and Poly(I:C) responses (**Figure 2A**). Pathways analysis [InnateDB (42)] identified an enrichment of cytokine and chemokine signalling pathways from upregulated genes in all responses, and IFN signalling pathways were prominent for imiquimod and Poly(I:C) CBMC responses (**Figures 2B**, **S1F**). Notably, the viral-related stimuli triggered a common set of 429 upregulated genes and this constituted a core antiviral response shared between Poly(I:C) and Imiquimod responses (**Figure S1G**). In addition, we identified 462 and 243 genes that were specifically upregulated in response to Poly(I:C) and Imiquimod respectively, demonstrating unique signalling pathways downstream of their receptors (**Figure S1G**). We next employed CIBERSORTx to estimate the post-culture cellular composition from the RNA-Seq data (43). Prominent cell types included monocytes, B cells, and CD4^+^ T cells (**Figures S2A,B**). The erythrocyte proportion was negligible as a result of immunomagnetic depletion (see Methods). Cell composition changes were identified between stimuli and age, but not sequence order or sex (**Figures S2C,D**). There was also no difference in the estimated cellular composition between individuals who were resistant or susceptible to sLRI in infancy, aligning with the baseline flow cytometry findings (**Figures S1C**, **S2D**). We also investigated variations in innate immune gene expression in the matching samples collected at birth versus age 5 (n=27 per age/stimuli) (**Figures S2E, F**). Interestingly, the LPS response at 5 years was characterised by upregulation of IFN-related genes, including *IRF1*, *STAT1*, and *IFIT1*-*3*, compared to birth. In contrast, IFN-related pathways were not prominent from differentially expressed genes between birth and age 5 following imiquimod or Poly(I:C) stimulation (**Figures S2E, F**). Finally, no genes were significantly different between individuals resistant and susceptible to sLRIs in infancy for any condition from this analysis (data not shown), suggesting that sLRI risk is not conferred by individual gene expression magnitude alone.

### Identification of co-expression networks underlying the innate immune responses at birth

**Figure 3:**
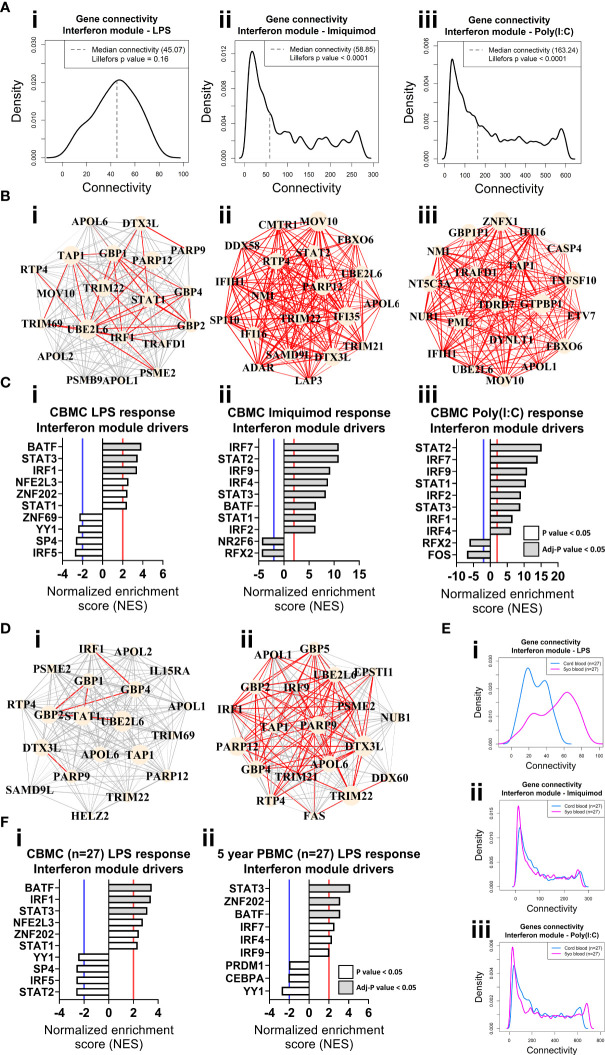
Network connectivity and master regulator analysis of IFN responses at birth and age 5 years identify IRF1 as a key driver. **(A)** Density plot of the LPS **(i)**, Imiquimod **(ii)**, and Poly(I:C) **(iii)** CBMC response IFN module connectivity, respectively. Dashed line denotes median. Lilliefors p value > 0.05 indicates normal distribution. **(B)** Network wiring diagrams of the top 20 most connected genes for the LPS **(i)**, Imiquimod **(ii)**, and Poly(I:C) **(iii)** CBMC IFN modules, respectively. Node size represents number of connections (degree) among the total network and edge width indicates strength of correlation (red edges > 0.8). **(C)** Top 10 master regulators for the respective CBMC IFN modules. Bar plots show normalized enrichment score (NES) for transcription factors which are significantly activated (NES>2, red line) or inactive/inhibited (NES<-2, blue line). Grey shading indicates an adjusted P value < 0.05. **(D)** Network wiring diagrams of the most connected CBMC LPS-induced IFN module genes from matched CBMC **(i)** and 5 year PBMC **(ii)** samples. Network characteristics are the same as above **(B). (E)** Network connectivity density plot for the interferon module gene connectivity of the matched CBMC (blue) and 5 year PBMC (magenta) responses to LPS **(i)**, Imiquimod **(ii)**, and Poly(I:C) **(iii)** stimulation. **(F)** Top drivers of the LPS-induced interferon module genes identified for matched CBMC **(i)** and 5 year PBMC (**ii**, 9 drivers were significant at P <0.05 samples. Bar plot characteristics are the same as above **(C)**.

Genes do not function in isolation, they work together in networks (57), and for this reason gene expression data is not only informative for differences in expression magnitude (e.g. fold changes) but also in network structure (e.g. connectivity). We employed weighted gene co-expression network analysis (WGCNA) to elucidate the global connectivity structure and functional organisation of gene expression patterns observed from our CBMC samples. This analysis identified 11, 11, and 8 co-expression modules for the LPS, Imiquimod and Poly(I:C) responses, respectively (**Figures 2C–E**, **S3A,B**). All responses exhibited upregulation of IFN and proinflammatory modules, and as we had already identified these as integral components of the cord blood innate responses with dimensionality reduction and differential expression analysis, they were therefore carried forward for downstream analysis (**Figures 2C–E**, **S3C–E**). The LPS response had the smallest IFN module (180 genes) compared to Imiquimod (1114 genes) and Poly(I:C) (2201 genes) and the inverse was true of the proinflammatory modules (LPS, 2297 genes; Imiquimod, 924 genes; Poly(I:C), 646 genes) (**Figure 2F**). Notably, there was substantial overlap between IFN and proinflammatory module genes of different stimuli, particularly between the Poly(I:C) IFN and LPS proinflammatory modules (n=385 genes) (**Figure S3F**). We next compared gene network patterns between the respective responses. First, we calculated module preservation statistics, and the results showed that the LPS-induced IFN module was highly preserved within the IFN modules of the imiquimod and Poly(I:C) responses but not vice versa (**Figure S3G**). The IFN modules associated with the imiquimod and Poly(I:C) responses were preserved within one another and the proinflammatory modules were preserved between all responses (**Figure S3G**). Second, we calculated ranked gene expression and ranked connectivity to compare modules. A prominent disparity was observed between expression magnitude (r = 0.88 & 0.82) and intra-module connectivity (r = 0.57 & 0.59) between the cord blood LPS-induced IFN module genes and the same genes following Imiquimod and Poly(I:C) stimulation, respectively (**Figure 2G**). To examine connectivity within modules, we plotted the connectivity density across all genes in each module and also identified the top 20 most connected genes (**Figures 3A, B**). The connectivity of the LPS-induced IFN module was characterised by a normal distribution, whereas the viral stimuli produced left-skewed distributions (**Figure 3A**). Key IFN signalling genes (e.g. *IRF1*, *STAT1*) were present among the most connected genes within the LPS-induced IFN module, however the strength of the most connected genes was reduced compared to the IFN modules of the viral stimuli (**Figure 3B**). The LPS-induced proinflammatory module displayed greater connectivity compared to the imiquimod- or Poly(I:C)-induced proinflammatory modules (**Figure S4A**). Genes encoding innate immune/proinflammatory cytokines (e.g. *IL1A/B*, *CXCL2/3/8*) were among to most connected genes in the proinflammatory modules of all responses at birth (**Figure S4A**). In summary, although viral nucleic acid and bacterial related stimuli activated overlapping sets of proinflammatory and IFN response genes, the underlying network structure was markedly different.

### Identification of master regulators of the innate immune responses at birth and age 5

We employed VIPER (50) analysis to identify master regulators which are predicted to drive module connectivity patterns. This approach revealed that the LPS-induced IFN module was putatively driven by BATF, STAT3 and IRF1 transcription factors (TFs) at birth, whereas the Imiquimod- and Poly(I:C)-induced IFN module top drivers included multiple STAT (e.g. STAT2) and IRF (e.g. IRF7) TFs (**Figure 3C**). The proinflammatory modules for all three responses were enriched for CEBPB, AP-1 (e.g. JUN, FOSL1/2) and NF-κB (e.g. NF-κB, RELB) (**Figure S4A**). Importantly, we repeated our analyses with input genes restricted to only those preserved from the LPS responses IFN (169/180, 93.89%) and proinflammatory (443/2297, 19.29%) modules and the result was unchanged (**Figures S4B, C**). Finally, we compared gene network patterns between CMBC and matched PBMCs samples (n=27) collected at 5 years. The connectivity of the genes of the LPS-induced IFN module was markedly higher at 5 years compared to birth among matched samples, suggesting that the wiring of this module is subject to developmental regulation (**Figures 3D, E**). Additionally, IRF1 enrichment was only identified from cord blood (**Figure 3F**). In contrast, the IFN responses provoked by imiquimod and Poly(I:C) stimulation displayed comparatively similar connectivity patterns between birth and age 5 years and, supporting this, the putative drivers were also comparable between birth and 5 years (e.g. STAT2, IRF7) (**Figures 3E**, **S5A**). Imiquimod and poly(I:C) proinflammatory modules were characterised by reduced intra-module connectivity in blood collected at 5 years compared to birth (**Figures S5B, C**).

### Innate immune responses at birth are predictive of sLRI in the first year of life

**Figure 4:**
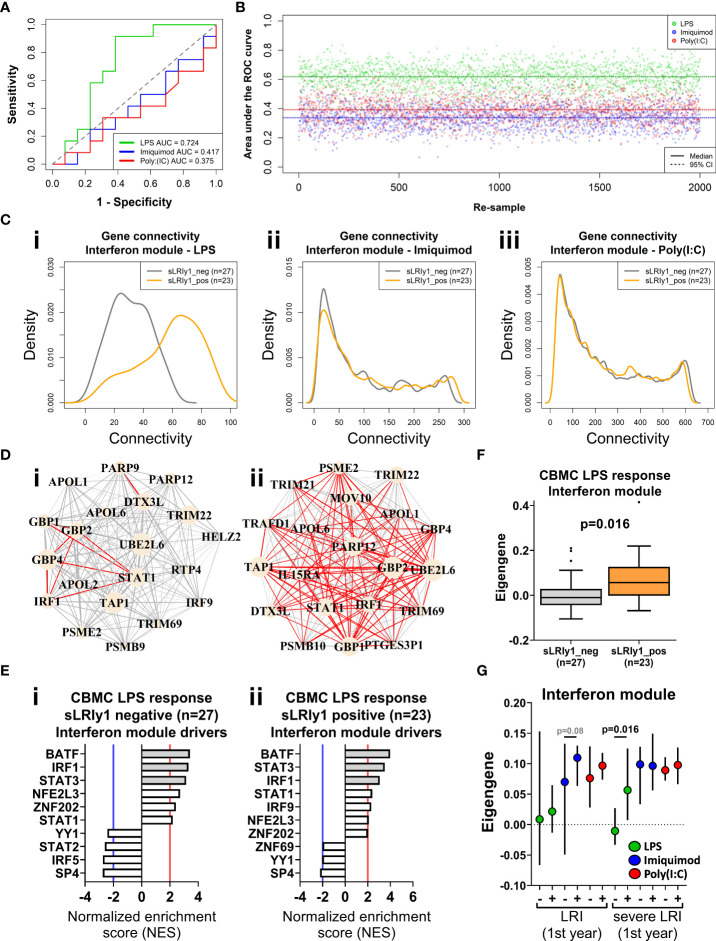
IFN genes activated following LPS stimulation at birth predict sLRI susceptibility in the first year of life. **(A)** Random forest (RF) classifiers were trained on the respective IFN module genes from half the study subjects and validated on the remaining subjects (50/50). RF models were optimised with respect to number of genes sampled at each split and number of trees grown. Plot depicts the area under the Receiver Operator Characteristic curve (AUC-ROC) defined by the rate of false (x-axis, 1-specificity) and true (y-axis, sensitivity) positives. **(B)** RF model predictions were repeated by re-sampling the training/validation set (50/50 random assignment) 2,000 times. Plot show the AUC-ROC for each re-sample, with median (solid lines) and 95% CIs (dashed lines). **(C)** Network connectivity density plot of IFN module gene networks stratified by individuals who did (orange) and did not (grey) record an sLRI in the first year of life. **(D)** Network wiring diagram of the most connected genes of the CBMC LPS-induced IFN module gene from individuals resistant (I, n=27) and susceptible (II n=23) to sLRIs in infancy. Node and edge characteristics are the same as **Figure 3B**. **(E)** Top 10 master regulators identified for the CBMC LPS-induced IFN response module for resistant (i) and susceptible (ii) subjects. Bar plot characteristics are the same **Figure 3C**. **(F)** Box-and-whisker plot of the CBMC LPS-induced IFN module eigengene, grouped by susceptible (orange) and resistant (grey) individuals. Boxes show median, 25^th^ and 75^th^ quartiles and whiskers are determined by the Tukey method; P value determined by Mann-Whitney U test. **(G)** Plot of IFN module eigengenes for CBMC responses grouped by individuals who were resistant (-) and susceptible (+) to LRIs and sLRIs in infancy. P values determined by Mann-Whitney U test and significant result reflects **(F)**. Plot shows median (symbol) and 95% CI (bars).

To determine whether innate immune responses at birth could predict the development of sLRIs in the first year of life, we randomly assigned the data set into training (50%, n=25) and validation sets (50%, n=25) and trained a random forest classifier on the CBMC IFN modules. The classifier trained on the LPS-induced IFN module genes could predict sLRIs in the first year of life with an accuracy of 72% in the validation data set (Area under the ROC curve = 0.724) (**Figure 4A**). Whilst the accuracy of this model may appear modest, it is known that risk biomarkers in general possess poor accuracy to predict subsequent disease over a specific time interval because the at-risk population will almost always be heterogeneous with respect to the disease outcome (58). In contrast, classifiers built from the Imiquimod- or Poly(I:C)-induced IFN module genes were not predictive of sLRIs in the first year of life (**Figure 4A**). To test whether this finding was reproducible given the relatively small sample numbers available as input, we repeated the analysis by randomly re-sampling subject membership in the training/validation sets (retaining the initial optimization parameters), and again found that only the LPS-induced IFN module genes could predict sLRIs in infancy better than chance on average (**Figures 4B**, **S6A–C**). Furthermore, we observed markedly different connectivity patterns for the LPS-induced IFN modules when stratified by individuals who did and did not experience and sLRI in the first year of life (**Figures 4C, D**, **S6D**), and this was not evident from the imiquimod- or Poly(I:C)-induced IFN modules (**Figures 4C**, **S6E, F**). Specifically, susceptible individuals had stronger gene network patterns for the LPS-induced IFN module, although the putative drivers of the response were comparable (IRF1, STAT3, BATF) (**Figures 4D, E**). Restricting the Imiquimod and Poly(I:C) IFN responses to only those genes of the LPS-induced IFN module did not exhibit noticeable differences in connectivity density patterns in relation to sLRI susceptibility in infancy (**Figures S6E(iii),F(iii)**). Whist the connectivity density plot of the LPS-induced IFN module of CBMCs of susceptible individuals (**Figure 4C**) resembled the overall connectivity density of the 5 year PBMC connectivity (n=27) (**Figure 3E**), the intra-module connectivity was significantly different (**Figures S6G, H**), suggesting the similarity may emerge from different processes. However, it should be noted that among the subjects which had a PBMC sample available at 5 years (n=27), the proportion of individuals who experienced an sLRI in infancy (29.63%) differed to that of the total subset (n=50, 46% sLRI positive in infancy). We also calculated module eigengenes to summarise overall module expression and compared this with clinical traits. The CBMC LPS-induced IFN module eigengene stratified individuals susceptible to sLRIs in the first year of life (p=0.016), as well as those with asthma (p=0.015) and current wheeze (p=0.02) at 5 years of age (**Figures 4F**, **S7A,B**). This result was only significant for the LPS response, was specific for the IFN module, and was only observed for comparisons of severe LRIs (**Figures 4G**, **S7C–E**).

### IFN responses induced in CBMCs by TLR ligands *in vitro* are representative of IFN responses during natural infections

**Figure 5:**
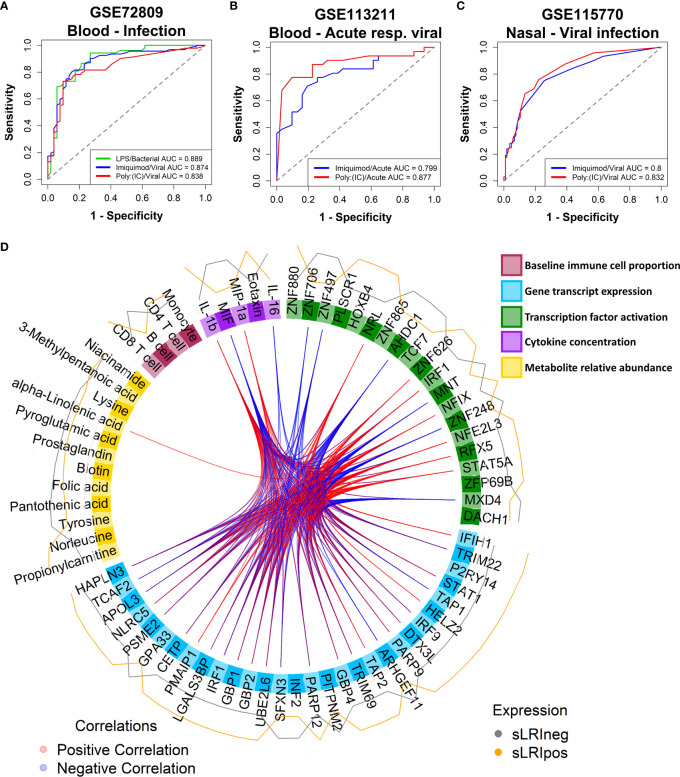
CBMC IFN responses reflect natural childhood response to infection; Multi-omic integration reinforces that LPS-induced IFN signalling at birth is a determinant of sLRI risk in infancy. **(A)** A random forest classifier was trained on Unstimulated and LPS or Imiquimod/Poly(I:C) CBMC IFN module gene expression data (n = 100) and used to predict children (<17yrs) hospitalized with bacterial (n = 52) and viral infections (n = 92), respectively, from healthy controls (n = 52) from blood-derived gene expression profiles. Gene expression data sets were restricted to available IFN module genes and RF models were optimised with respect to the number of genes and trees. Plot depicts the AUC-ROC defined by the rate of false (x-axis, 1-specificity) and true (y-axis, sensitivity) positives. **(B)** RF classifiers trained on Unstimulated and Imiquimod or Poly(I:C) CBMC IFN module gene expression data (n=100) and used to predict PBMC gene expression profiles from infants (<18mo; n=15) and children (18mo-5yrs; n=16) presenting to hospital with acute viral respiratory infections from profiles collected during convalescence. Plot depicts the AUC-ROC. **(C)** RF classifiers trained on Unstimulated and Imiquimod or Poly(I:C) CBMC IFN module gene expression data (n=100) and used to predict asthmatic children (6-17yrs) with cold-like symptoms who do (n=193) and do not (n=105) have detectable airway viral infection from nasal-derived gene expression profiles. Plot depicts the AUC-ROC. **(D)** Circos plot displaying the multi-layer risk profile for sLRI susceptibility in infancy determined from multi-omic data integration, showing between block correlation from the 1^st^ latent component; correlations stronger that ±0.8 are shown. Peripheral lines represent the relative expression of features from individuals who were resistant (grey) or susceptible (orange) to sLRIs in the first year of life. Input data was adjusted with respect to matched unstimulated samples (except baseline immunophenotype data).

We questioned whether the IFN module gene expression profiles exhibited by CBMCs following *in vitro* culture with model antigens in our study are reflective of naturally occurring IFN responses to childhood infections *in vivo*. To address this issue, we trained RF classifiers on our CBMC-derived IFN module genes and used them to classify samples from publicly available data sets from the Gene Expression Omnibus. Gene expression data from external cohorts was filtered to only those genes present in the corresponding IFN modules for each analysis. The first data set comprised whole blood gene expression profiles from children (<17yrs) with febrile illnesses requiring hospitalization with confirmed bacterial (n = 52) or viral (n = 92) infections versus healthy controls (n = 52) [GSE72809 (59)]. We found that RF classifiers trained on LPS- and Imiquimod/Poly(I:C)-induced IFN module genes accurately predicted children with bacterial (AUC = 0.889) and viral (AUC = 0.874/0.838) infections, respectively (**Figures 5A**, **S7F**). The second data set consisted of PBMC samples from infants (<18mo, n=30) and young children (18mo-5yrs, n = 32) who were hospitalized with acute viral bronchiolitis [GSE113211 (60)]. Classifiers built on unstimulated and either Imiquimod- (AUC=0.8) or Poly(I:C)- (AUC=0.877) induced IFN genes could accurately stratify samples collected during acute illness compared to matched post-convalescent samples (symptom-free, 8.8 ± 2.5 weeks post-infection), independent of age (**Figures 5B**, **S7G**). The models performed well for infants (AUC = 0.922, Poly(I:C); AUC = 0.827, Imiquimod) and children (AUC = 0.789, Poly(I:C); AUC = 0.842, Imiquimod) separately (**Figure S7G**). The third data set consisted of nasal-derived gene expression profiles from study visits of asthmatic children (6-17yrs) with viral-related or non-viral “cold”-like illness (1-6 days post-onset), some of which later experienced exacerbations (n=83, 58 were viral-positive) [GSE115770 (61)]. Symptomatic children with respiratory viral infections were accurately predicted from symptomatic, yet virus-negative, children from Imiquimod (AUC=0.8) and Poly(I:C) (AUC=0.832) defined RF classifiers (**Figures 5C**, **S7H**). Additionally, there was comparable accuracy classifying virus-positive and virus-negative asthmatic children who subsequently experienced an exacerbation (within 10 days of symptom onset) (**Figure S7H**). In the same study, prediction performance was less accurate from peripheral blood-derived gene expression profiles (**Figure S7H**). Taken together, these analyses demonstrate that CBMC-derived IFN gene expression patterns induced with LPS, Imiquimod, or poly(I:C) in this study are representative of childhood IFN responses to microbial pathogens.

### Multi-omic integration of LPS-stimulated CBMC data

**Figure 6:**
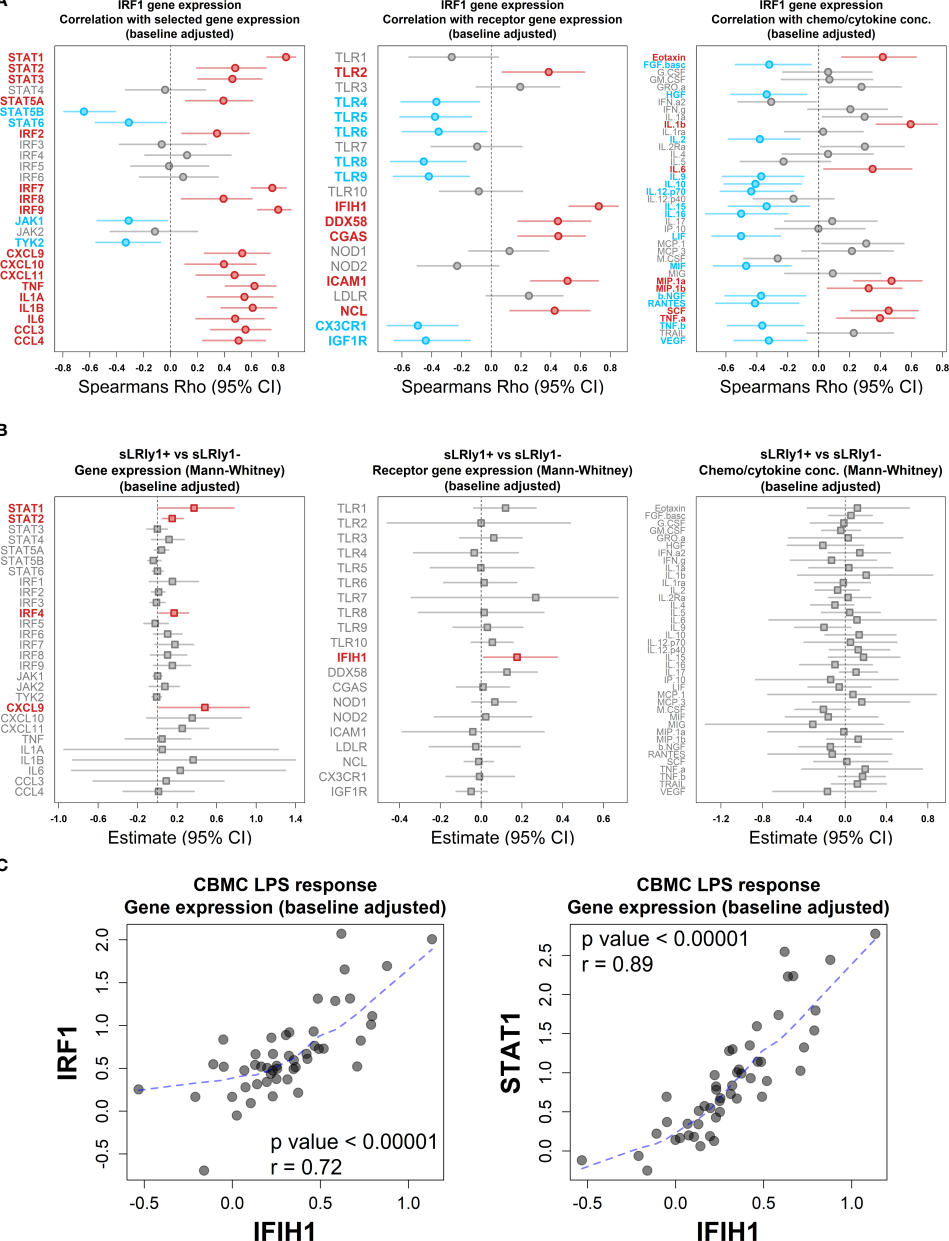
LPS-induced IRF1 correlates with key interferon/proinflammatory mediators. **(A)** Plot of the association between LPS-induced IRF1 gene expression with selected IFN and proinflammatory gene expression (left), viral-related receptor gene expression (center) and chemo/cytokine protein concentration (right). Data was adjusted with respect to matched unstimulated samples and plots shows Spearman’s Rho value (symbol) and 95% CI (bars, 1000 bootstraps); Red and blue data points/labels denote positive and negative correlations, respectively, with a BH-adjusted p value <0.05. **(B)** Analysis of selected IFN and proinflammatory gene expression (left), viral-related receptor gene expression (center) and chemo/cytokine protein concentration (right) with respect to sLRI susceptibility in the first year of life. Data was adjusted with respect to matched unstimulated samples and plots show the Mann-Whitney U test estimates and 95% CIs for CBMC data of individuals who are susceptible compared to resistant to sLRIs in infancy. Red data points/labels indicate increased expression with a p value < 0.05. **(C)** Spearman’s correlation and associated p value between IFIH1 (x-axis) and IRF1/STAT1 (y-axis) gene expression from CBMC samples stimulated with LPS (n=50). Data was adjusted with respect to matched unstimulated samples. Dashed blue line represents a loess fit of the data.

Lastly, we employed multi-omic data integration (DIABLO (52)) to identify correlated molecular features across biological layers which may confer sLRI risk. Input data consisted of CBMC baseline immune cell type proportions (n = 8), significantly variable mRNA transcripts (n=6344), VIPER-derived regulon activity scores (n = 1224), metabolites (n = 49), and cytokine/chemokine proteins (n = 39). Importantly for this analysis, input genes were selected as those which were significantly variable between LPS-stimulated and unstimulated CBMC samples and were not selectively enriched for IFN-related transcripts. The data reinforced that LPS-induced IFN-signalling transcripts (*IRF9*, *STAT1*, *GBP2/4*) and IRF1 activity were key determinants of risk for sLRI in the first year of life, in combination with lymphocyte and monocyte proportions, immune regulators (e.g. RFX5, NFIX), amino acids, and proinflammatory cytokines/chemokines (IL-1β, MIP-1α, MIF) (**Figure 5D**). As we separately identified LPS-induced IRF1 activity from network, master regulator, and integrative analyses, we further investigated IRF1 gene expression correlations. *IRF1* gene expression at birth positively correlated with selective STAT and IRF family transcription factor genes (e.g. *STAT1*, *IRF7/9*), proinflammatory mediators (e.g. IL-1β, IL-6, CCL3/MIP-1α), and viral-related receptor genes (e.g. *ICAM1*, *IFIH1*) (**Figure 6A**). Additionally, CBMC *STAT1* and *IFIH1* gene expression was higher in response to LPS among individuals who were susceptible to sLRIs in infancy, and *IFIH1* expression correlated with *IRF1* and *STAT1* expression (**Figures 6B, C**).

## Discussion

Severe viral lower respiratory tract infections (sLRIs) are a leading cause of hospitalization for infants and children and constitute a major risk factor for subsequent asthma development (2–6). Whilst it is increasingly recognised that bacterial and viral pathogens may interact to drive the pathogenesis of sLRIs, the underlying innate immune mechanisms are not well understood. We employed a multi-omic approach to systematically profile innate immune responses to bacterial (LPS) and viral nucleic acid (Poly(I:C)/Imiquimod) related stimuli at birth to first characterize these responses and then investigate whether any response patterns are associated with susceptibility to sLRI in the first year of life. The data showed that whilst innate immune responses to the panel of stimuli comprised overlapping proinflammatory and IFN-mediated gene expression programs, the LPS but not Poly(I:C)/Imiquimod response profiles at birth were predictive of sLRI incidence in the first year of life. Moreover, sLRI susceptibility was associated with the activation of a network of IFN genes, and the connectivity patterns of this network in cord blood LPS responses were strikingly exaggerated among infants susceptible to sLRI. Furthermore, the connectivity pattern of these genes was highly variable between the cord and 5 year LPS responses. These findings were specific for the LPS-induced IFN responses and were not observed following activation of viral nucleic acid sensing pathways, nor from proinflammatory module genes of any response tested, suggesting that the wiring of the LPS response is specifically altered in children who are at heightened risk for sLRI in infancy. It is noteworthy that expression of the LPS-induced IFN module was not associated with mild (non-wheezy/non-febrile) lower respiratory tract infections, highlighting a specific link to infection severity. Master regulator analysis identified IRF1 as a key driver of LPS-induced IFN responses at birth. By age 5, the data showed that the activity of IRF1 may be replaced by other members of the IRF transcription factor family, including IRF7, suggesting that this response is subject to developmental regulation. In contrast, IRF7 was the dominant driver of Poly(I:C)-/Imiquimod-induced IFN response at birth and 5 years. IRF1 was also identified as a highly connected node of the LPS-induced IFN network and correlated with distinct IFN-signalling (e.g. *STAT1* but not *JAK1/TYK2*) and proinflammatory (e.g. *CXCL9/10/11*, IL-1β) mediators, several of which exhibited significantly higher expression in infants at risk of sLRI. These data suggest that an LPS-induced and IRF1-regulated IFN gene network, detectable at birth, is associated with sLRI susceptibility in infancy. Consequently, we conclude that susceptibility to sLRI in infancy may be in part already determined at birth and this may be exploited to identify at-risk infants for early intervention and identify potential targets for drug development.

The contribution of bacteria and their products to the severity of viral-related respiratory infections has been suggested by numerous studies. For example, environmental LPS exposure modulates the severity of RSV infections depending on the levels of LPS exposure and TLR4 genotype (62). Moreover, studies from our group in the same cohort have demonstrated that sLRIs are often preceded by the transient incursion of pathogenic bacteria in the airway microbiome (17, 18). Multiple other studies have reported that the presence of pathogenic bacteria in the airways is associated with more severe viral respiratory tract infections for both RSV and RV (63–66). Additionally, bacterial colonization of the airway in neonates with *Streptococcus pneumoniae*, *Haemophilus influenzae*, or *Moraxella catarrhalis* was associated with persistent wheeze and severe exacerbations of wheeze (15), which are generally initiated by viral respiratory infections. Finally, Illi et al. demonstrated that LPS responses at 12 months of age in individuals who carry asthma-risk alleles on 17q21 are associated with risk of wheeze (32).

The proposition that heightened LPS-induced IFN responses/gene network connectivity patterns at birth may confer risk of viral-related sLRI during infancy at first sight may appear counterintuitive given the acknowledged protective role of IFNs in antiviral immunity (67, 68). However, hyper-production of IFNs in the airways during viral-associated infections, especially during infancy, are also known to contribute to accompanying inflammatory symptom severity (60, 69). Furthermore, IFN responses during bacterial infections have pleiotropic effects which may be beneficial or detrimental, depending on the site of infection and the specific pathogen involved (70, 71). For example, type I IFN mediated suppression of IL-1β responses (72) on the one hand attenuates lethal hyperinflammation associated with S. *pyogenes* (73) and on the other hand diminishes the antimicrobial function of IL-1β, resulting in increased airway and systemic M. *tuberculosis* colonisation (74). This suggests a balance exists between IFN and proinflammatory responses which impacts the clinical outcome of bacterial infection, although it is not clear how anti-bacterial responses may protect against or exacerbate viral infections. IRF1 promotes the constitutive expression of interferon-mediated antiviral programs at baseline and the inducible expression of these programs triggered by respiratory viral infections (75–78), and acts as a branch point between IFN responses and induction of specific pro-inflammatory genes (79). The function of IRF1 following bacterial infections is incompletely understood, although it appears essential for IFN-related inflammasome activation during *Francisella novicida* infection (75, 80) indicating a role in IFN and proinflammatory responses following pathogenic bacterial exposure. It is also notable in this context that IRF1 gene variants have been linked to childhood asthma risk and dysregulated proinflammatory responses (81). We did not observe a direct difference in IRF1 expression between individuals who did or did not record an sLRI in the first year of life at the time point investigated in our study. This suggests that the sLRI risk putatively associated with IRF1 may be conferred by its regulatory actions rather than its gene expression magnitude, or that IRF1 expression dysregulation occurs earlier than was measured in this study (18hrs). It is our interpretation of the data that IRF1 is a key driver of the LPS-induced IFN response networks associated with sLRI susceptibility in the first year of life.

The immune system of newborns is subject to drastic developmental changes in the first weeks (82) and months (83) of life. Since our study focused on CBMC-derived innate immune responses, we explored the extent to which CBMC responses reflect immune responses to infections occurring at later ages during childhood by applying classifiers trained on gene expression data generate in this study to infection-associated host response data derived from published cohorts. This approach was not intended as validation of the principal findings linking LPS-induced IFN response at birth with subsequent sLRI risk, but rather to establish whether the IFN response networks characterized from our *in vitro* experiments in CBMCs are representative of those operating in nature. LPS-induced IFN responses from CBMCs were used to accurately stratify children presenting to hospital with current bacterial infections, compared to controls, from whole blood samples (59). Likewise, Imiquimod/Poly(I:C)-induced CBMC IFN responses accurately classified children with febrile viral infections. Moreover, the CBMC-derived IFN responses induced by imiquimod or Poly(I:C) could classify infants and children with viral bronchiolitis and asthma exacerbations from blood and airway samples compared to controls, suggesting that these signatures are robust to some extent to variations in cellular composition between circulating blood and airway tissue. We also found that the accuracy of the random forest models was higher when predicting infants (<18mo) compared to younger (18mo-5yrs) (GSE113211 (60)) or older (6-17yrs) children (GSE115770 (61)). These data support that the IFN gene networks identified from our *in vitro* investigation of cord blood are *bona fide* response mediators of infection in real world contexts.

We acknowledge that our study has limitations that should be addressed. Firstly, gene expression profiles were generated from mixed cord blood cell populations and as such cannot distinguish cell-specific information. Further investigation with single-cell RNA sequencing may localize gene expression programs potentially responsible for sLRI risk in individual cells. Secondly, the study population consisted of 50 subjects from a high-risk cohort, limiting the power to detect disease-associated mechanisms. Follow-up studies with samples from large, unselected cohorts may identify more subtle mechanisms that confer risk for sLRI. Additionally, we utilised CBMC samples for this work, because they are readily available and abundant at birth. However, recent advances in sample processing methods now enable the generation of multi-omic data from small sample volumes, enabling longitudinal profiling of infants/children with natural infections (82). We profiled innate immune responses at a single timepoint (18hrs), and therefore our analyses cannot capture response dynamics. Finally, we employed three TLR ligands to mimic PRR activation events experienced during bacterial or viral infections. However, we acknowledge using TLR ligands is not equivalent to using live bacteria or virus. Notwithstanding these limitations, the major strengths of this study lie in the systems biology approach that provided genome-wide investigation of the CBMC responses, and the well characterized prospective cohort design, which allowed us to investigate sLRI risk with the totality of viral infections and relevant clinical outcomes recorded. In summary, our findings demonstrate that the risk of sLRI in early life is in part already determined at birth, and that the developmental status of LPS-induced interferon responses may represent a key factor which confers susceptibility. Our findings provide a rationale for the early identification of infants at risk for sLRI and identifies potential targets which may be relevant for drug development.

## Data availability statement

The datasets presented in this study are available. RNA-Seq data is available from the NCBI Gene Expression Omnibus repository with accession number GSE184383. Raw and processed cytokine/chemokine data are provided as a Supplement (**Data S1**). Raw and processed metabolite data are provided as a Supplement (**Data S2**).

## Ethics statement

Ethics was approved by The University of Western Australia (reference RA/4/1/7560). Written informed consent to participate in this study was provided by the participants’ legal guardian/next of kin.

## Author contributions

PGH, AB, and PDS conceived the study and designed the experiments. JFR analysed the data. JFR and MS assisted with the experimental design and optimized and performed the experiments. JFR, MS, AB, SNR, DM, BJH, DIB, and DHS were involved in data acquisition. JFR, AB, PGH, DHS, SNR, DIB, MC, and YVK were involved in determining the analysis approach and optimization. JFR, AB, and PGH interpreted the data and drafted the manuscript. All authors contributed to the article and approved the submitted version.

## Funding

This work was funded by National Health and Medical Research Council (NHMRC) project grant #1129996. PDS is a Leadership Fellow (L3) of the NHMRC.

## Acknowledgments

The authors would like to thank the study participants and their families for their involvement.

## Conflict of interest

JFR and AB are co-inventors on a provisional patent filed subsequent to this work. JFR and AB are co-founders, equity holders, and directors of a startup company Respiradigm Pty Ltd related to this provisional patent.

The remaining authors declare that the research was conducted in the absence of any commercial or financial relationships that could be construed as a potential conflict of interest.

## Publisher’s note

All claims expressed in this article are solely those of the authors and do not necessarily represent those of their affiliated organizations, or those of the publisher, the editors and the reviewers. Any product that may be evaluated in this article, or claim that may be made by its manufacturer, is not guaranteed or endorsed by the publisher.

## Supplementary material

Data S1 Raw and normalized cytokines multiplex assay data.

Data S2 Raw and corrected metabolite relative abundances.
