# Supplementary material for: Lipopolysaccharide-induced interferon response networks at birth are predictive of severe viral lower respiratory infections in the first year of life
Source: Front Immunol. 2022 Aug 5;13:876654. doi: 10.3389/fimmu.2022.876654 (PMC9389556; doi:10.3389/fimmu.2022.876654)
Supplement: Data S1 — Raw and normalized cytokines multiplex assay data. [file DataSheet_1.zip › Supplementary Material/Supplementary Methods.PDF]

Table S1. Differences in key characteristics of the subset stratified by the primary outcome.

	sLRIy1 negative (n=27)	sLRIy1 positive (n=23)	OR (95% CI)	P value
**Sex (female)**	14/27 (51.85%)	10/23 (43.48%)	0.72 (0.2-2.51)	0.58
**Gestation (weeks; mean [range])**	39.11 [36-41]	39.17 [37-41]	NA	0.96
**Birth weight (grams; mean [range])**	3468.56 [2755-4270]	3529.35 [2920-4415]	NA	0.65
**SPT⁺ at 0.5, 2, or 5 years**	16/27 (59.26%)	14/23 (60.87%)	1.07 (0.3-3.88)	1
**URI⁺ in first year**	26/27 (96.3%)	21/23 (86.96%)	0.41 (0.01-8.41)	0.59
**No. of URIs in first year (mean [range])**				
**LRI⁺ in first year**	16/27 (59.26%)	23/23 (100%)	Inf (2.97-Inf)	>0.001
**No. of LRIs in first year (mean [range])**				
**Any sLRI⁺ in first year (wheezy and/or febrile)**	0/27 (0%)	23/23 (100%)	Inf (77.85-Inf)	>0.0001
**No. of sLRIs in first year (mean [range])**	0 [0-0]	1.91 [1-5]	NA	>0.0001
**Only wheezy sLRI⁺ in first year**	0/27 (0%)	7/23 (30.43%)	Inf (2.05-Inf)	0.002
**Only febrile sLRI⁺ in first year**	0/27 (0%)	8/23 (34.78%)	Inf (2.56-Inf)	>0.001
**Wheezy and febrile sLRI⁺ in first year**	0/27 (0%)	8/23 (34.78%)	Inf (2.56-Inf)	>0.001
**Current wheeze at 5 years**	6/25 (24%)	8/18 (44.44%)	2.48 (0.57-11.49)	0.2
**Asthma at 5 years**	4/19 (21.05%)	5/15 (33.33%)	1.57 (0.28-9.39)	0.71
**Never wheeze (years 3-5)**	13/25 (52%)	8/18 (44.44%)	0.86 (0.25-2.8)	1
**Viral detection per individual in any infectious episode in the first year of life (mean [range])**				
**Rhinovirus**	1.7 [0-5]	1.96 [0-5]	NA	0.52
**Resp. Syncytial virus**	0.33 [0-1]	0.52 [0-2]	NA	0.26
**Parainfluenza**	0.26 [0-1]	0.3 [0-1]	NA	0.73
**Coronavirus**	0.26 [0-1]	0.3 [0-2]	NA	0.93
**Influenza**	0.11 [0-1]	0.3 [0-4]	NA	0.51
**Adenovirus**	0.04 [0-1]	0.22 [0-1]	NA	0.055
**Metapneumovirus**	0.15 [0-1]	0 [0-0]	NA	0.06
**Total**	2.85 [0-6]	3.61 [0-10]	NA	0.26

Abbreviations: CAS=Childhood Asthma Study, OR=Odds Ratio, CI=Confidence Interval, URI=Upper respiratory Infection (viral), (s)LRI=(severe) Lower respiratory Infection (viral), SPT=Skin Prick Test. sLRIy1 represents the primary outcome (sLRI incidence in the first year of life). For categorical

variables, odds ratios, 95% CIs and accompanying P values were determined by Fishers Exact test. For continuous variables, P values were determined by Mann-Whitney U test. Variation in participant number relates to data availability (see Methods). Related to Table 1.

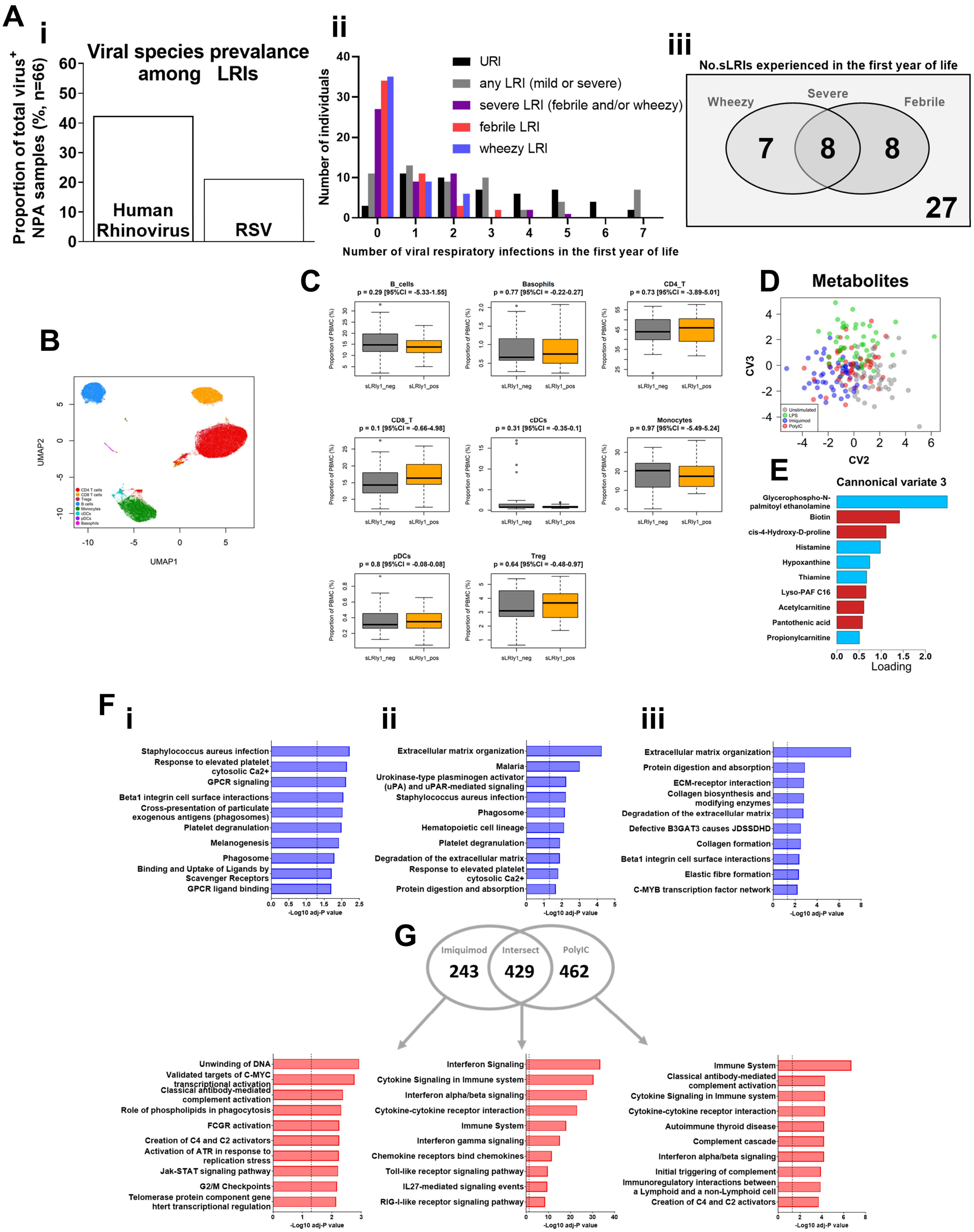
A
i
Viral species prevalance among LRIs
Proportion of total virus+ NPA samples (%, n=66)
Human Rhinovirus
RSV
ii
URI
any LRI (mild or severe)
severe LRI (febrile and/or wheezy)
febrile LRI
wheezy LRI
Number of individuals
Number of viral respiratory infections in the first year of life
iii
No.sLRIs experienced in the first year of life
Wheezy
Severe
Febrile
7
8
8
27
B
UMAP1
UMAP2
C
B_cells
p = 0.29 [95%CI = -5.33-1.55]
Basophils
p = 0.77 [95%CI = -0.22-0.27]
CD4_T
p = 0.73 [95%CI = -3.89-5.01]
CD8_T
p = 0.1 [95%CI = -0.66-4.98]
cDCs
p = 0.31 [95%CI = -0.35-0.1]
Monocytes
p = 0.97 [95%CI = -5.49-5.24]
pDCs
p = 0.8 [95%CI = -0.08-0.08]
Treg
p = 0.64 [95%CI = -0.48-0.97]
Proportion of PBMC (%)
sLRIy1_neg
sLRIy1_pos
D
Metabolites
CV2
CV3
Unstimulated
LPS
Imiquimod
PolyIC
E
Cannonical variate 3
Glycerophospho-N-palmitoyl ethanolamine
Biotin
cis-4-Hydroxy-D-proline
Histamine
Hypoxanthine
Thiamine
Lyso-PAF C16
Acetylcarnitine
Pantothenic acid
Propionylcarnitine
Loading
F
i
Staphylococcus aureus infection
Response to elevated platelet cytosolic Ca2+
GPCR signaling
Beta1 integrin cell surface interactions
Cross-presentation of particulate exogenous antigens (phagosomes)
Platelet degranulation
Melanogenesis
Phagosome
Binding and Uptake of Ligands by Scavenger Receptors
GPCR ligand binding
-Log10 adj-P value
ii
Extracellular matrix organization
Malaria
Urokinase-type plasminogen activator (uPA) and uPAR-mediated signaling
Staphylococcus aureus infection
Phagosome
Hematopoietic cell lineage
Platelet degranulation
Degradation of the extracellular matrix
Response to elevated platelet cytosolic Ca2+
Protein digestion and absorption
-Log10 adj-P value
iii
Extracellular matrix organization
Protein digestion and absorption
ECM-receptor interaction
Collagen biosynthesis and modifying enzymes
Degradation of the extracellular matrix
Defective B3GAT3 causes JDSSDHD
Collagen formation
Beta1 integrin cell surface interactions
Elastic fibre formation
C-MYB transcription factor network
-Log10 adj-P value
G
Imiquimod
243
Intersect
429
PolyIC
462
Unwinding of DNA
Validated targets of C-MYC transcriptional activation
Classical antibody-mediated complement activation
Role of phospholipids in phagocytosis
FCGR activation
Creation of C4 and C2 activators
Activation of ATR in response to replication stress
Jak-STAT signaling pathway
G2/M Checkpoints
Telomerase protein component gene htert transcriptional regulation
-Log10 adj-P value
Interferon Signaling
Cytokine Signaling in Immune system
Interferon alpha/beta signaling
Cytokine-cytokine receptor interaction
Immune System
Interferon gamma signaling
Chemokine receptors bind chemokines
Toll-like receptor signaling pathway
IL27-mediated signaling events
RIG-I-like receptor signaling pathway
-Log10 adj-P value
Immune System
Classical antibody-mediated complement activation
Cytokine Signaling in Immune system
Cytokine-cytokine receptor interaction
Autoimmune thyroid disease
Complement cascade
Interferon alpha/beta signaling
Initial triggering of complement
Immunoregulatory interactions between a Lymphoid and a non-Lymphoid cell
Creation of C4 and C2 activators
-Log10 adj-P value

Figure S1. LRI respiratory virus prevalence and additional analysis of baseline flow cytometry, metabolomic CVA, and transcriptomic pathways. (**A**) (i) Bar plot of the proportion of human Rhinovirus and Respiratory Syncytial Virus (RSV) detected from infectious NPAs collected during episodes of LRI in the study subset. (ii) Number of respiratory infections in the first year of life for the study population (n=50), stratified by viral infection category. The x-axis shows the number of viral infections and the y-axis shows the number of individuals. Different viral infection categories are denoted by colour, and each individual is represented in each category. (iii) Venn diagram displaying the distribution of severe LRIs, split into wheezy, febrile, and wheezy and febrile. 27 individuals from the study subset did not experience an sLRI in the first year of life. (**B**) Uniform Manifold Approximation and Projection (UMAP) dimensionality reduction of flow cytometric data of immune cell markers. Plot shows coordinates of the first (x-axis) and second (y-axis) UMAP components and depicts relative cell type proportion and clustering of 100,000 cells randomly selected from all subjects. (**C**) Box plots of the proportions of identified immune cell types grouped by individuals who did (n=23, orange) and did not (n=27, grey) record a sLRI in infancy. Box plots show median, 25th and 75th quartiles, ±1.5xIQR, and outliers; p values and 95% CIs defined by Mann-Whitney U test. (**D**) Plot of cross-validated (2000 permutations) canonical variates 2 and 3 coordinates for the metabolomic data set (related to Figure 1C(iii)). (**E**) Horizontal bar plots showing top contributing features of cross validated canonical variate 3 of the metabolomic data; corresponds to y-axis of Figure S1D (above) (related to Figures 1C(iii) & D(iii & vi)). (**F**) Top 10 overrepresented pathways from significantly downregulated genes of the CBMC LPS (i), Imiquimod (ii), and Poly(I:C) (iii) responses compared to matched unstimulated controls, respectively. X-axis shows the Benjamini-Hochberg (FDR) corrected overrepresentation p value ($-Log_{10}$ transformed); black dashed line indicated corrected $p \approx 0.05$ (related to Figure 2B). (**G**) Venn diagram of the number of intersect and unique genes upregulated following Imiquimod and Poly(I:C) stimulation of CBMC, and their associated top 10 most overrepresented pathways (related to figures 2A and 2B).

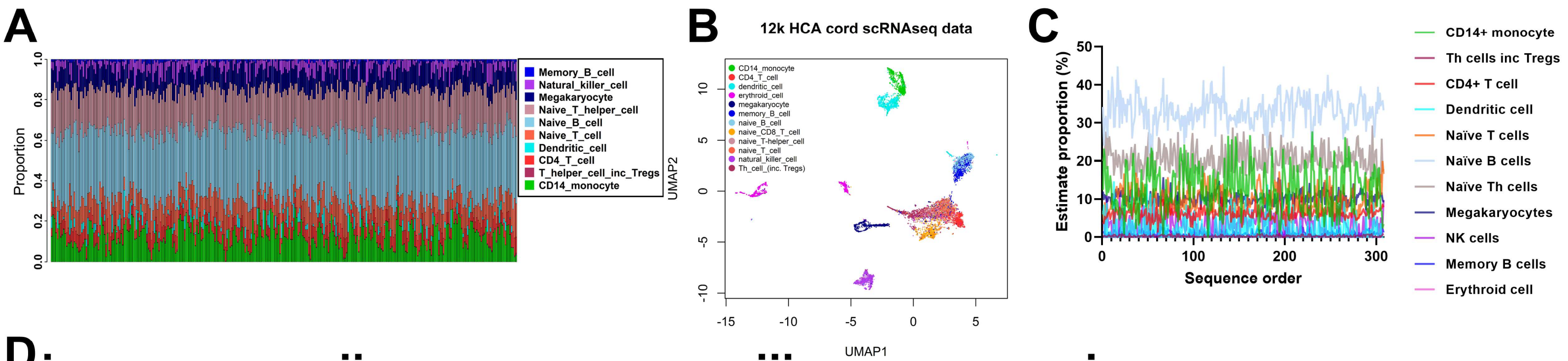

D

i

Stimuli comparison

	LPS	Imiquimod	Poly(I:C)
CD14+ Monocyte	5.47, 8.55	1.24, 4.24	-0.93, 1.66
Th cells inc Treg	0.04, 0.15	0.12, 0.22	0.17, 0.29
CD4+ T cell	-0.02, 0.62	0.57, 1.46	0.71, 1.46
Dendritic cell	-5.43, -2.77	-7.18, -4.9	-6.2, -3.78
Naive T cell	-3.18, -1.73	-1.28, 0.2	-1.7, 0.1
Naive B cell	-1.98, 0.37	-0.11, 2.36	-1.1, 1.65
Naive Th cell	0.7, 2.53	2.45, 4.69	3.09, 5.27
Megakayrocyte	-1.66, -0.72	-0.53, 0.19	-1.4, -0.4
NK cell	-1.49, -0.24	-3.51, -2.16	-2.04, 0.39
Memory B cell	-0.28, 0.05	-0.4, -0.09	-0.2, 0.36

ii

Cord vs 5yr comparison

	Unstimulated	LPS	Imiquimod	Poly(I:C)
CD14+ Monocyte	-5.31, -0.81	-11.16, -7.04	-8.81, -3.08	-9.35, -5.12
Th cells inc Treg	-0.01, 0.23	0.19, 0.43	-0.05, 0.12	0.08, 0.32
CD4+ T cell	-0.41, 0.92	0.06, 1.22	0.21, 1.73	-0.87, 0.71
Dendritic cell	-1.54, 2.37	-1.74, 1.18	-1.63, 2.01	-1.86, 1.77
Naive T cell	-0.12, 2.73	2.22, 4.14	0.21, 2.76	2.8, 6.08
Naive B cell	-1.88, 2.22	-0.4, 3.02	0.04, 3.63	-1.45, 2.96
Naive Th cell	-1.45, 0.73	-0.93, 1.9	-0.87, 2.08	-2.92, -0.17
Megakayrocyte	-0.74, 0.52	-0.73, 0.65	0.27, 1.61	-0.91, 0.73
NK cell	-0.36, 1.9	1.1, 3.61	-1.67, 1.34	0.31, 4.9
Memory B cell	0.47, 1.02	0.23, 0.72	0, 0.58	0.19, 1.28

iii

Sex comparison

	Unstimulated	LPS	Imiquimod	Poly(I:C)
CD14+ Monocyte	-1.09, 3.23	-3.47, 2.18	-3.4, 2.6	-2.02, 3.28
Th cells inc Treg	0, 0.07	-0.16, 0	-0.12, 0.04	-0.18, 0.02
CD4+ T cell	-0.99, 0.34	-0.66, 0.72	-0.74, 0.5	-1.23, 0.32
Dendritic cell	-3.8, 0.84	-1.06, 0.11	0, 0	0, 0.4
Naive T cell	-1.81, 0.92	-1.5, 1.28	-1.12, 1.66	-1, 2.34
Naive B cell	-2.21, 2.1	-2.71, 2.75	-2.52, 1.65	-3.86, 1.02
Naive Th cell	-0.1, 2.56	-0.41, 2.9	-2.26, 1.67	-2.53, 1.51
Megakayrocyte	-0.95, 0.41	-0.94, 0.76	-0.66, 0.85	-0.95, 0.53
NK cell	-0.97, 1.34	-0.97, 1.13	0, 1.6	-0.97, 2.6
Memory B cell	-0.19, 0.29	-0.41, 0.16	-0.32, 0.15	-0.45, 0.28

iv

sLRI year 1 comparison

	Unstimulated	LPS	Imiquimod	Poly(I:C)
CD14+ Monocyte	-0.64, 3.98	-0.32, 4.82	-0.71, 5.21	-0.85, 4.08
Th cells inc Treg	0, 0.01	-0.08, 0.04	-0.05, 0.11	-0.12, 0.1
CD4+ T cell	-0.72, 0.59	-0.66, 0.9	-0.72, 0.69	-1.52, 0.15
Dendritic cell	-0.67, 3.54	-0.21, 1.11	0, 0	0, 0.51
Naive T cell	-2.7, -0.02	-1.77, 1.23	-2.54, -0.23	-1.99, 1.23
Naive B cell	-3.69, 0.16	-4.97, -0.17	-3.94, 0.37	-3.51, 1.25
Naive Th cell	-2.15, 0.5	-1.12, 2.12	0.17, 3.53	-1.98, 2
Megakayrocyte	-1.2, 0.17	-1.58, -0.28	-1.27, 0.24	-1.2, 0.32
NK cell	-1.12, 1.25	-0.57, 1.62	-1.64, 0.36	-0.74, 2.92
Memory B cell	-0.15, 0.3	-0.3, 0.24	-0.34, 0.14	-0.41, 0.37

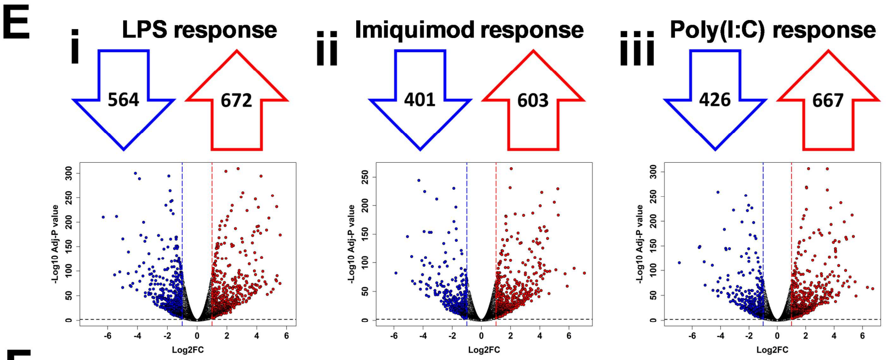

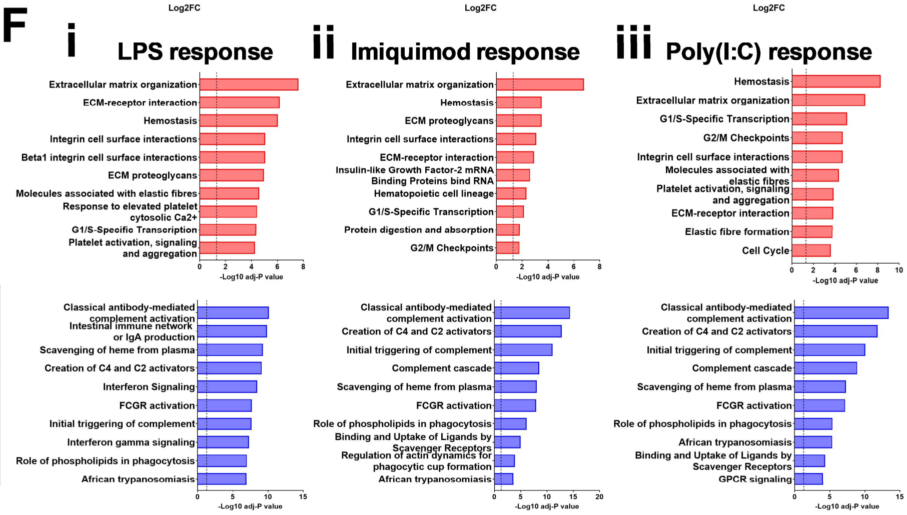

iv

Symbol	logFC	FDR
IFNG	-1.841	3.45E-28
IFIT3	-1.779	3.29E-166
TLR3	-1.644	1.93E-16
IFIT1	-1.566	1.78E-75
OASL	-1.482	7.88E-58
IFIT2	-1.232	4.03E-82
MX1	-1.097	3.56E-107
OAS1	-1.097	8.10E-79
STAT1	-1.045	1.48E-89
IRF1	-1.044	3.05E-97

Figure S2. Estimation of cellular composition from post-culture gene expression profiles and additional IFN gene expression signature identified from LPS stimulation of 5yr PBMC. (**A**) Stacked bar plot of cellular proportion of immune cells estimated by CIBERSORTx. Y-axis indicates the proportion (%) of each cell type of the total estimated cell types. (**B**) UMAP plot of the 1000 randomly selected scRNAseq cord blood cell profiles from each cell type. Plot shows coordinates of the first (x-axis) and second (y-axis) UMAP components, and colors indicate the individual cell annotation, as determined by the original investigators. (**C**) Plot of estimate immune cell proportions as a function of sequence order. (**D**) Results from between group comparisons of CIBERSORTX determined cellular proportions for unstimulated versus stimulated CBMC (paired) (**i**), CBMC versus 5yr PBMC (matched samples, paired) (**ii**), sex difference in CBMC proportion (unpaired) (**iii**), and CBMC proportion differences between individuals resistant/susceptible to sLRIs in infancy (unpaired) (**iv**). Each cell in a corresponding table represents the 95% CI (low to high) of a Wilcoxon SRT (pseudo-median) or a Mann-Whitney U (difference in location) sample estimate for paired and unpaired tests, respectively. Cells shaded red denote an associated FDR-adjusted P value < 0.01. (**E**) Results of differential expression analysis between match CBMC and 5yr PBMC samples, adjusted for baseline expression. Arrows indicate the number of upregulated (red) and downregulated (blue) genes identified for each analysis; Log_2-fold change > 1, BH adjusted-P value < 0.01. Volcano plots show differential gene expression patterns of the corresponding responses. (**F**) Top 10 most significantly overrepresented pathways among the upregulated (red, top row) and downregulated (blue, bottom roe) genes for differentially expressed between CBMCs and 5yr PBMCs for the LPS (i), Imiquimod (ii) and Poly(I:C) (iii) responses. Insert (iv) shows genes, and their differential expression characteristics, responsible for interferon pathway overrepresentation following LPS treatment of 5yr PBMC compared to matched CMBC (figure S1F (i, bottom)). Related to Figure 2.

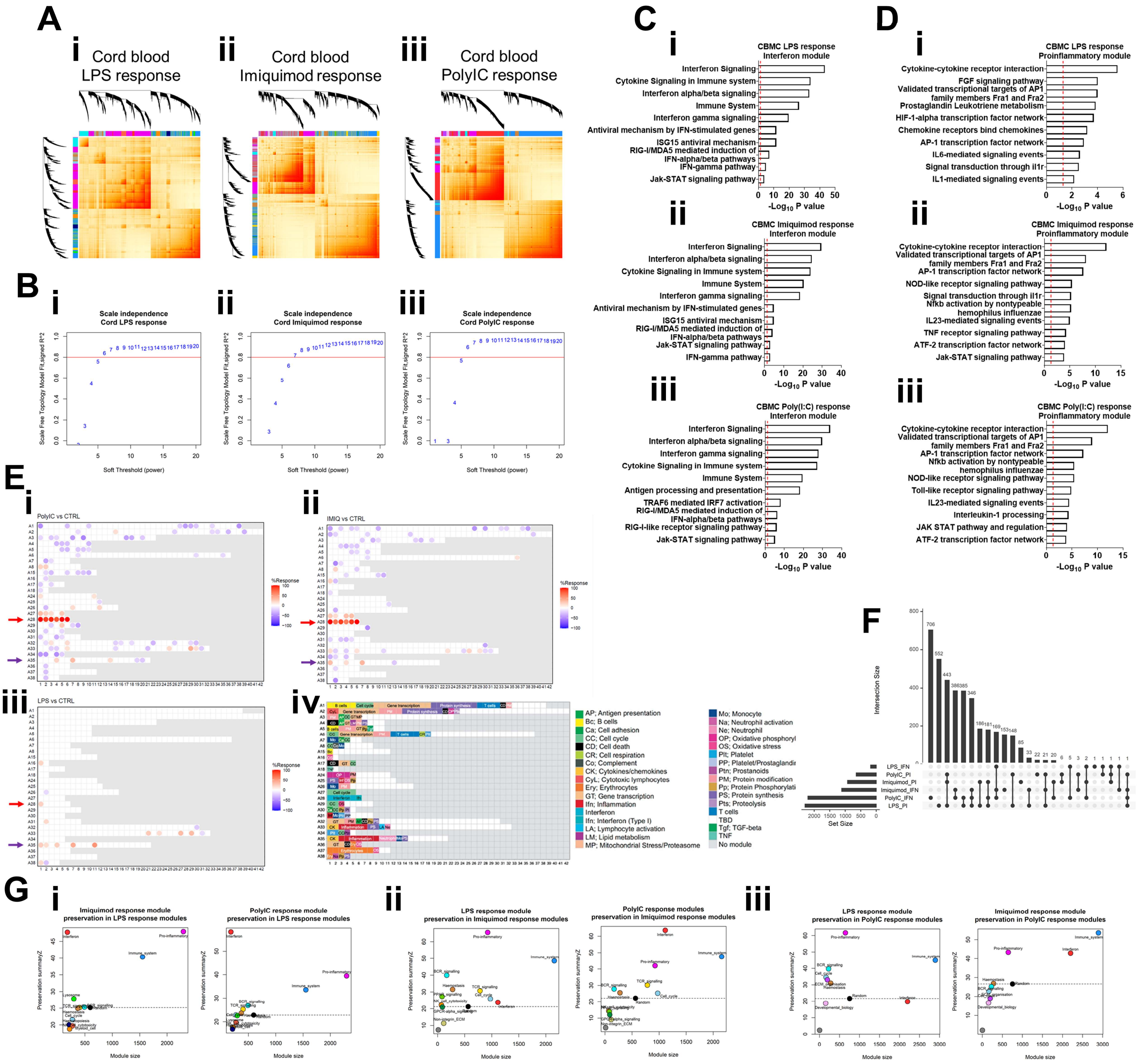

A
i Cord blood LPS response
ii Cord blood Imiquimod response
iii Cord blood PolyIC response
B
Scale independence Cord LPS response
Scale independence Cord Imiquimod response
Scale independence Cord PolyIC response
Scale Free Topology Model Fit,signed R^2
Soft Threshold (power)
C
i CBMC LPS response Interferon module
Interferon Signaling
Cytokine Signaling in Immune system
Interferon alpha/beta signaling
Immune System
Interferon gamma signaling
Antiviral mechanism by IFN-stimulated genes
ISG15 antiviral mechanism
RIG-I/MDA5 mediated induction of IFN-alpha/beta pathways
IFN-gamma pathway
Jak-STAT signaling pathway
-Log10 P value
ii CBMC Imiquimod response Interferon module
Interferon Signaling
Interferon alpha/beta signaling
Cytokine Signaling in Immune system
Immune System
Interferon gamma signaling
Antiviral mechanism by IFN-stimulated genes
ISG15 antiviral mechanism
RIG-I/MDA5 mediated induction of IFN-alpha/beta pathways
Jak-STAT signaling pathway
IFN-gamma pathway
-Log10 P value
iii CBMC Poly(I:C) response Interferon module
Interferon Signaling
Interferon alpha/beta signaling
Interferon gamma signaling
Cytokine Signaling in Immune system
Immune System
Antigen processing and presentation
TRAF6 mediated IRF7 activation
RIG-I/MDA5 mediated induction of IFN-alpha/beta pathways
RIG-I-like receptor signaling pathway
Jak-STAT signaling pathway
-Log10 P value
D
i CBMC LPS response Proinflammatory module
Cytokine-cytokine receptor interaction
FGF signaling pathway
Validated transcriptional targets of AP1 family members Fra1 and Fra2
Prostaglandin Leukotriene metabolism
HIF-1-alpha transcription factor network
Chemokine receptors bind chemokines
AP-1 transcription factor network
IL6-mediated signaling events
Signal transduction through il1r
IL1-mediated signaling events
-Log10 P value
ii CBMC Imiquimod response Proinflammatory module
Cytokine-cytokine receptor interaction
Validated transcriptional targets of AP1 family members Fra1 and Fra2
AP-1 transcription factor network
NOD-like receptor signaling pathway
Signal transduction through il1r
Nfkb activation by nontypeable hemophilus influenzae
IL23-mediated signaling events
TNF receptor signaling pathway
ATF-2 transcription factor network
Jak-STAT signaling pathway
-Log10 P value
iii CBMC Poly(I:C) response Proinflammatory module
Cytokine-cytokine receptor interaction
Validated transcriptional targets of AP1 family members Fra1 and Fra2
AP-1 transcription factor network
Nfkb activation by nontypeable hemophilus influenzae
NOD-like receptor signaling pathway
Toll-like receptor signaling pathway
IL23-mediated signaling events
Interleukin-1 processing
JAK STAT pathway and regulation
ATF-2 transcription factor network
-Log10 P value
E
i PolyIC vs CTRL
ii IMIQ vs CTRL
iii LPS vs CTRL
%Response
iv
AP; Antigen presentation
Bc; B cells
Ca; Cell adhesion
CC; Cell cycle
CD; Cell death
CR; Cell respiration
Co; Complement
CK; Cytokines/chemokines
CyL; Cytotoxic lymphocytes
Ery; Erythrocytes
GT; Gene transcription
Ifn; Inflammation
Interferon
In; Interferon (Type I)
LA; Lymphocyte activation
LM; Lipid metabolism
MP; Mitochondrial Stress/Proteasome
Mo; Monocyte
Na; Neutrophil activation
Ne; Neutrophil
OP; Oxidative phosphoryl
OS; Oxidative stress
Plt; Platelet
PP; Platelet/Prostaglandi
Ptn; Prostanoids
PM; Protein modification
Pp; Protein Phosphorylati
PS; Protein synthesis
Pts; Proteolysis
T cells
TBD
Tgf; TGF-beta
TNF
No module
F
Intersection Size
Set Size
LPS_IFN
PolyIC_PI
Imiquimod_PI
Imiquimod_IFN
PolyIC_IFN
LPS_PI
G
i Imiquimod response module preservation in LPS response modules
PolyIC response module preservation in LPS response modules
ii LPS response module preservation in Imiquimod response modules
PolyIC response modules preservation in Imiquimod response modules
iii LPS response module preservation in PolyIC response modules
Imiquimod response module preservation in PolyIC response modules
Preservation summaryZ
Module size

Figure S3. Identification of co-expression networks (WGCNA) underlying innate immune function; Topological overlap, soft power, enrichment, module overlap, and preservation. Related to Figure 2. (**A**) Topological Overlap Matrix (TOM) plots of the LPS (i), Imiquimod (ii), and Poly(I:C) (iii) CBMC responses, respectively. Dendrograms show gene clustering and row/column colours denote modules. Module colours relate to those shown in Figure 2C-D. Red colour in the TOM plot indicates stronger gene pair correlation. (B) Elbow plot of soft threshold power used for the LPS (i), Imiquimod (ii), and Poly(I:C) (iii) CBMC responses, respectively. Plot shows the soft threshold power (x-axis) and the coefficient of determination (R^2, y-axis) of the model fitted for that power. (**C**) (running downwards) Ten selected significantly overrepresented pathways (InnateDB) for genes contained within the IFN module of the CBMC responses to the LPS (i), Imiquimod (ii), and Poly(I:C) (iii), respectively. (**D**) (running downwards) Same analysis as above (Figure S3C) for the respective proinflammatory modules. (**E**) Module fingerprint grid plot showing the results BloodGen3Modulefor the Poly(I:C) vs control (i), Imiquimod vs control (ii), and LPS vs control (iii). Each module is in a fixed position, and red (blue) dots represent modules in which member genes are predominantly increased (decrease) from the group-wise comparison. Rows are organised in “Aggregates” of modules which are considered functionally related module sets. The red arrow indicated “Aggregate 28” which is associated with interferon-related modules, and the purple arrow indicate “Aggregate 35” which is associate with inflammation-related modules. (iv) Key depicting annotated modules, reproduced from Figure 2 of the original publication by Rinchai et. al. in *Bioinformatics,* Volume 37, Issue 16, 15 August 2021, Pages 2382–2389(1). (**F**) Matrix-based layout plot (Upset plot) showing intersections and number of genes of the IFN and proinflammatory module genes for the LPS Imiquimod, and Poly(I:C) CBMC responses. (**G**) Plots of module size (x-axis) and module preservation summary Z statistic (y-axis) for the LPS (i), Imiquimod (ii), and Poly(I:C) (iii) responses compared to each other response. Points on the plots represent modules for the respective response. The black symbol and associated dashed line in each plot indicate the summary Z statistic calculated from randomly sampled genes across modules (n=mean module size) and modules plotted above this line are considered preserved between the corresponding modules of the analysis.

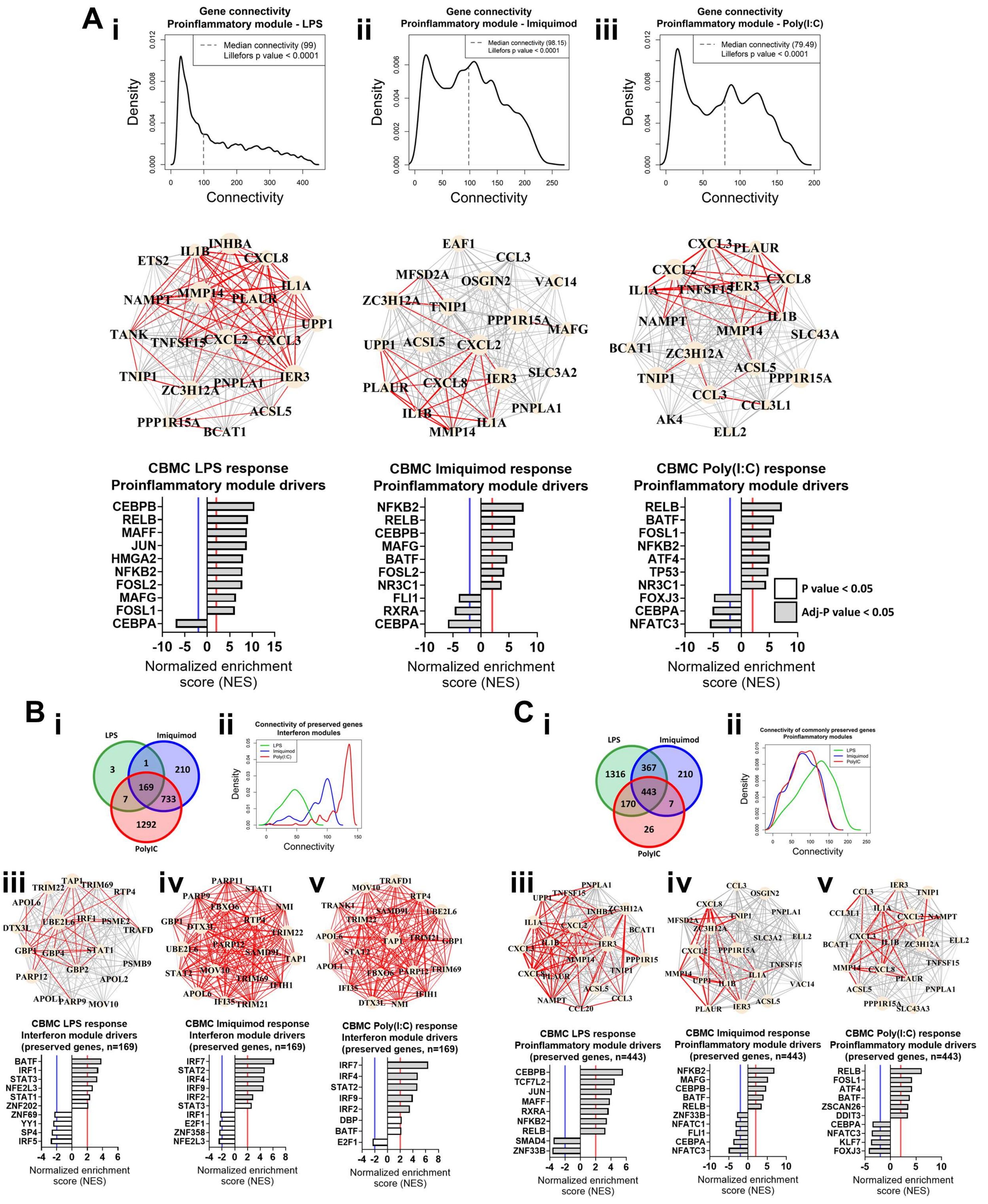
A i
Gene connectivity
Proinflammatory module - LPS
Median connectivity (99)
Lillefors p value < 0.0001
Density
Connectivity
ii
Gene connectivity
Proinflammatory module - Imiquimod
Median connectivity (98.15)
Lillefors p value < 0.0001
iii
Gene connectivity
Proinflammatory module - Poly(I:C)
Median connectivity (79.49)
Lillefors p value < 0.0001
CBMC LPS response
Proinflammatory module drivers
CEBPB
RELB
MAFF
JUN
HMGA2
NFKB2
FOSL2
MAFG
FOSL1
CEBPA
CBMC Imiquimod response
Proinflammatory module drivers
NFKB2
RELB
CEBPB
MAFG
BATF
FOSL2
NR3C1
FLI1
RXRA
CEBPA
CBMC Poly(I:C) response
Proinflammatory module drivers
RELB
BATF
FOSL1
NFKB2
ATF4
TP53
NR3C1
FOXJ3
CEBPA
NFATC3
P value < 0.05
Adj-P value < 0.05
Normalized enrichment score (NES)
B i
LPS
Imiquimod
PolyIC
ii
Connectivity of preserved genes
Interferon modules
iii
iv
v
CBMC LPS response
Interferon module drivers
(preserved genes, n=169)
BATF
IRF1
STAT3
NFE2L3
STAT1
ZNF202
ZNF69
YY1
SP4
IRF5
CBMC Imiquimod response
Interferon module drivers
(preserved genes, n=169)
IRF7
STAT2
IRF4
IRF9
IRF2
STAT3
IRF1
E2F1
ZNF358
NFE2L3
CBMC Poly(I:C) response
Interferon module drivers
(preserved genes, n=169)
IRF7
IRF4
STAT2
IRF9
IRF2
DBP
BATF
E2F1
C i
ii
Connectivity of commonly preserved genes
Proinflammatory modules
iii
iv
v
CBMC LPS response
Proinflammatory module drivers
(preserved genes, n=443)
CEBPB
TCF7L2
JUN
MAFF
RXRA
NFKB2
RELB
SMAD4
ZNF33B
CBMC Imiquimod response
Proinflammatory module drivers
(preserved genes, n=443)
NFKB2
MAFG
CEBPB
BATF
RELB
ZNF33B
NFATC1
FLI1
CEBPA
NFATC3
CBMC Poly(I:C) response
Proinflammatory module drivers
(preserved genes, n=443)
RELB
FOSL1
ATF4
BATF
ZSCAN26
DDIT3
CEBPA
NFATC3
KLF7
FOXJ3

Figure S4. Network characteristics and drivers of innate CBMC proinflammatory responses. Related to Figure 3. (**A**) Network and master regulator analysis of the LPS (i), Imiquimod (ii), and Poly(I:C) (iii) CBMC response proinflammatory modules, respectively. Panels run from top to bottom for each response and relate to Figure 3A-C. Top row; Density plot of the respective response proinflammatory module connectivity. Dashed lines denote median connectivity (grey). Lilliefors p value > 0.05 indicate normally distributed connectivity. Middle row; Network wiring diagrams of the top 20 most connected genes for the respective CBMC proinflammatory modules, respectively. Node size represents number of connections (degree) among the total network and edge with indicates strength of connection (red edges denote a correlation > 0.8). Bottom row; Top 10 master regulators identified by VIPER analysis for the respective CBMC proinflammatory modules. Bar plots show normalized enrichment score (NES) for transcription factors which are significantly activate (NES>2, red line) or inactive/inhibited (NES<-2, blue line). Grey shading indicates an adjusted P value < 0.05. (**B**) Analysis of gene connectivity of IFN module genes common to the LPS, Imiquimod, and Poly(**I**:C) CBMC responses (n=169). (**i**) Venn diagram of the overlap of IFN module genes from the three CBMC responses. (ii) Density plots of the gene connectivity for the 169 common IFN genes. (**iii-v**) Network wiring diagrams of the top 20 most connected of the 169 genes for the respective CBMC responses (top row) and top 10 master regulators identified that drive gene expression of these genes (bottom row) for the LPS (**iii**), Imiquimod (**iv**), and Poly(**I**:C) (**v**), respectively. (**C**) Analysis of gene connectivity of proinflammatory module genes common to the LPS, Imiquimod, and Poly(**I**:C) CBMC responses (n=443). This panel reflects Figure S4B for the proinflammatory modules.

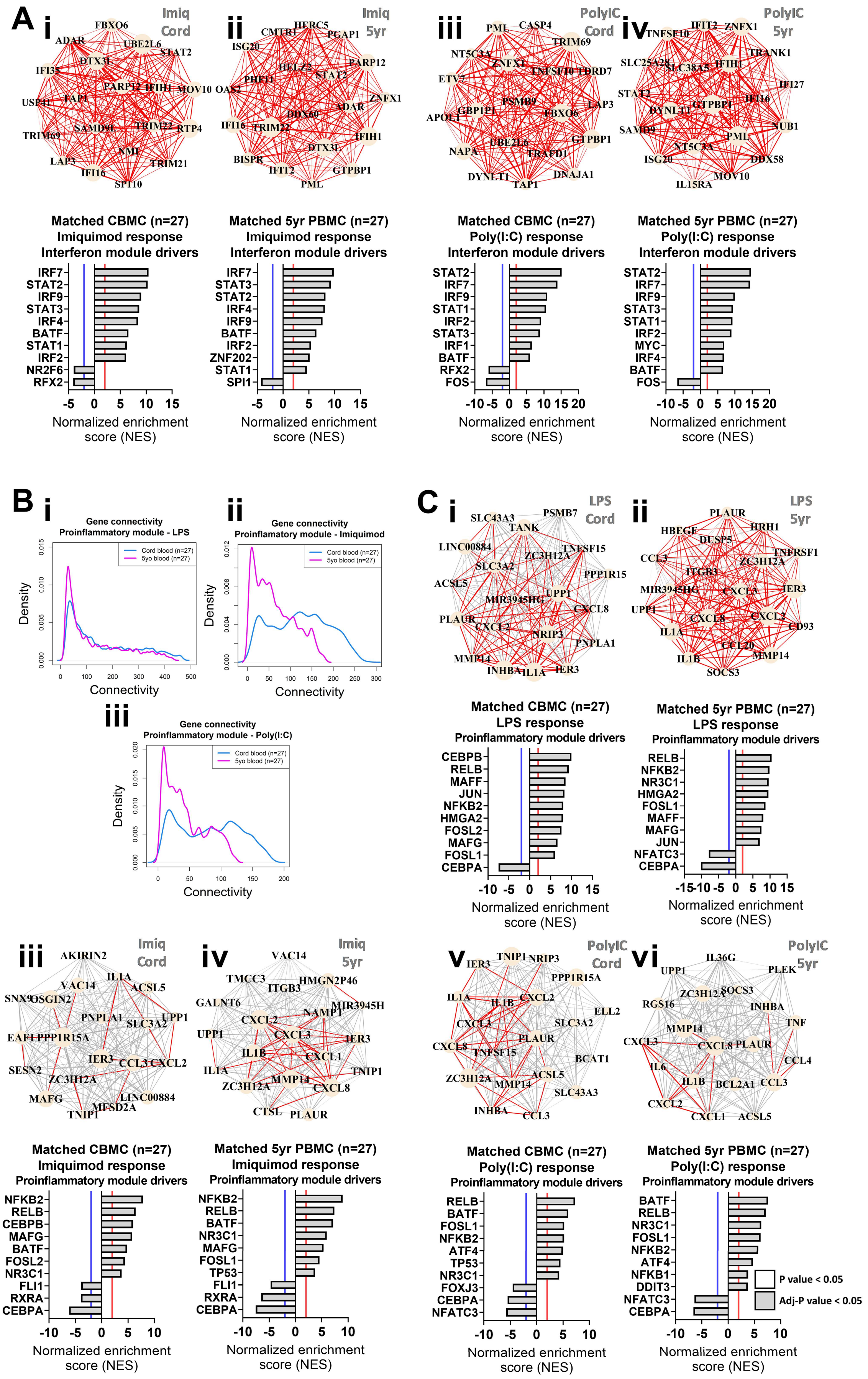
A
i
Imiq Cord
ii
Imiq 5yr
iii
PolyIC Cord
iv
PolyIC 5yr
Matched CBMC (n=27) Imiquimod response Interferon module drivers
IRF7
STAT2
IRF9
STAT3
IRF4
BATF
STAT1
IRF2
NR2F6
RFX2
Matched 5yr PBMC (n=27) Imiquimod response Interferon module drivers
IRF7
STAT3
STAT2
IRF4
IRF9
BATF
IRF2
ZNF202
STAT1
SPI1
Matched CBMC (n=27) Poly(I:C) response Interferon module drivers
STAT2
IRF7
IRF9
STAT1
IRF2
STAT3
IRF1
BATF
RFX2
FOS
Matched 5yr PBMC (n=27) Poly(I:C) response Interferon module drivers
STAT2
IRF7
IRF9
STAT3
STAT1
IRF2
MYC
IRF4
BATF
FOS
Normalized enrichment score (NES)
B
i
Gene connectivity Proinflammatory module - LPS
ii
Gene connectivity Proinflamatory module - Imiquimod
iii
Gene connectivity Proinflammatory module - Poly(I:C)
Cord blood (n=27)
5yo blood (n=27)
Density
Connectivity
C
i
LPS Cord
ii
LPS 5yr
Matched CBMC (n=27) LPS response Proinflammatory module drivers
CEBPB
RELB
MAFF
JUN
NFKB2
HMGA2
FOSL2
MAFG
FOSL1
CEBPA
Matched 5yr PBMC (n=27) LPS response Proinflammatory module drivers
RELB
NFKB2
NR3C1
HMGA2
FOSL1
MAFF
MAFG
JUN
NFATC3
CEBPA
iii
Imiq Cord
iv
Imiq 5yr
v
PolyIC Cord
vi
PolyIC 5yr
Matched CBMC (n=27) Imiquimod response Proinflammatory module drivers
NFKB2
RELB
CEBPB
MAFG
BATF
FOSL2
NR3C1
FLI1
RXRA
CEBPA
Matched 5yr PBMC (n=27) Imiquimod response Proinflammatory module drivers
NFKB2
RELB
BATF
NR3C1
MAFG
FOSL1
TP53
FLI1
RXRA
CEBPA
Matched CBMC (n=27) Poly(I:C) response Proinflammatory module drivers
RELB
BATF
FOSL1
NFKB2
ATF4
TP53
NR3C1
FOXJ3
CEBPA
NFATC3
Matched 5yr PBMC (n=27) Poly(I:C) response Proinflammatory module drivers
BATF
RELB
NR3C1
FOSL1
NFKB2
ATF4
NFKB1
DDIT3
NFATC3
CEBPA
P value < 0.05
Adj-P value < 0.05
Normalized enrichment score (NES)

Figure S5. Network characteristics and drivers of innate CBMC IFN responses to Imiquimod/Poly(I:C) and CBMC/5yr PBMC proinflammatory responses. Related to Figure 3. (**A**) Wiring diagrams of top 20 connected genes and drivers of the IFN modules for the matched CBMC and 5yr PBMC (n=27) Imiquimod and Poly(I:C) responses. (left-right) CBMC imiquimod (i), 5yr PBMC Imiquimod (ii), CBMC Poly(I:C) (iii), and 5yr PBMC Poly(I:C) (iv) responses. Horizontal bar plots represent top 10 master regulators of the responses. Wiring diagrams and bar plot characteristics are the same as Figure 3B and 3C, respectively. (**B**) Gene connectivity density plots of the LPS (i), Imiquimod (ii), and Poly(I:C) (iii) CBMC (blue) and 5yr PBMC (magenta) proinflammatory modules, respectively. (**C**) Wiring diagrams of top 20 connected genes and drivers of the proinflammatory modules for the matched CBMC and 5yr PBMC (n=27) LPS (i, ii), Imiquimod (iii, iv) and Poly(I:C) (v, vi) responses. Wiring diagrams and bar plot characteristics are the same as Figure 3B and 3C, respectively.

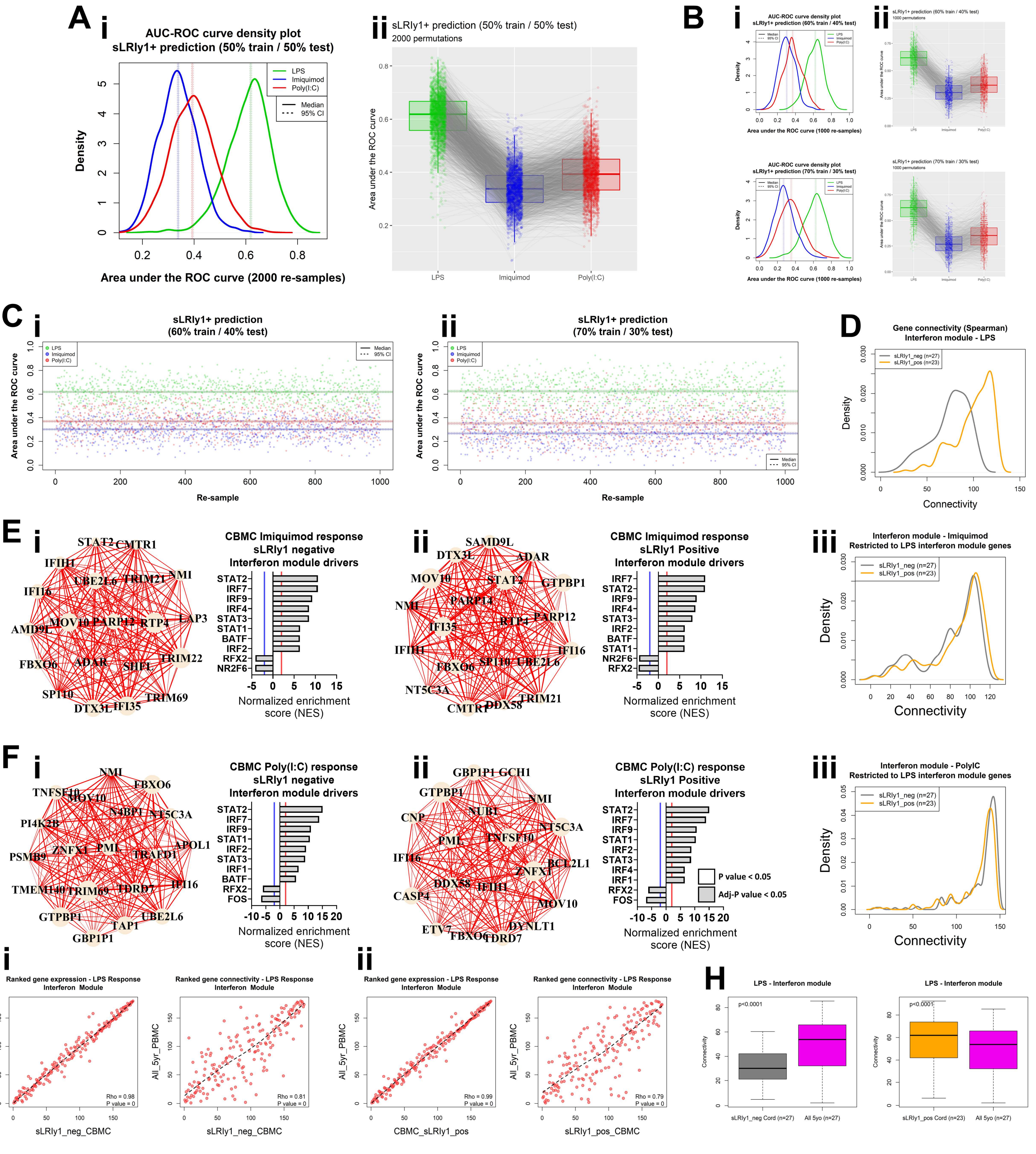
A
i
AUC-ROC curve density plot
sLRly1+ prediction (50% train / 50% test)
LPS
Imiquimod
Poly(I:C)
Median
95% CI
Density
Area under the ROC curve (2000 re-samples)
ii
sLRly1+ prediction (50% train / 50% test)
2000 permutations
Area under the ROC curve
LPS
Imiquimod
Poly(I:C)
B
i
AUC-ROC curve density plot
sLRly1+ prediction (60% train / 40% test)
AUC-ROC curve density plot
sLRly1+ prediction (70% train / 30% test)
Area under the ROC curve (1000 re-samples)
ii
sLRly1+ prediction (60% train / 40% test)
1000 permutations
sLRly1+ prediction (70% train / 30% test)
1000 permutations
C
i
sLRly1+ prediction
(60% train / 40% test)
ii
sLRly1+ prediction
(70% train / 30% test)
Area under the ROC curve
Re-sample
D
Gene connectivity (Spearman)
Interferon module - LPS
sLRly1_neg (n=27)
sLRly1_pos (n=23)
Density
Connectivity
E
i
CBMC Imiquimod response
sLRly1 negative
Interferon module drivers
STAT2
IRF7
IRF9
IRF4
STAT3
STAT1
BATF
IRF2
RFX2
NR2F6
Normalized enrichment score (NES)
ii
CBMC Imiquimod response
sLRly1 Positive
Interferon module drivers
IRF7
STAT2
IRF9
IRF4
STAT3
IRF2
BATF
STAT1
NR2F6
RFX2
Normalized enrichment score (NES)
iii
Interferon module - Imiquimod
Restricted to LPS interferon module genes
sLRly1_neg (n=27)
sLRly1_pos (n=23)
Density
Connectivity
F
i
CBMC Poly(I:C) response
sLRly1 negative
Interferon module drivers
STAT2
IRF7
IRF9
STAT1
IRF2
STAT3
IRF1
BATF
RFX2
FOS
Normalized enrichment score (NES)
ii
CBMC Poly(I:C) response
sLRly1 Positive
Interferon module drivers
STAT2
IRF7
IRF9
STAT1
IRF2
STAT3
IRF4
IRF1
RFX2
FOS
P value < 0.05
Adj-P value < 0.05
Normalized enrichment score (NES)
iii
Interferon module - PolyIC
Restricted to LPS interferon module genes
sLRly1_neg (n=27)
sLRly1_pos (n=23)
Density
Connectivity
G
i
Ranked gene expression - LPS Response
Interferon Module
All_5yr_PBMC
sLRly1_neg_CBMC
Rho = 0.98
P value = 0
Ranked gene connectivity - LPS Response
Interferon Module
Rho = 0.81
P value = 0
ii
Ranked gene expression - LPS Response
Interferon Module
CBMC_sLRly1_pos
Rho = 0.99
P value = 0
Ranked gene connectivity - LPS Response
Interferon Module
sLRly1_pos_CBMC
Rho = 0.79
P value = 0
H
LPS - Interferon module
p<0.0001
Connectivity
sLRly1_neg Cord (n=27)
All 5yo (n=27)
LPS - Interferon module
p<0.0001
sLRly1_pos Cord (n=23)
All 5yo (n=27)

Figure S6. Random forest re-sampling confirms sLRI susceptibility prediction accuracy of LPS-induced IFN module genes; comparative network and driver characteristics of the primary outcome for Imiquimod and Poly(I:C) responses. Related to Figure 4. (**A**) IFN module gene random forest model predictions were repeated by re-sampling the training/validation set (50/50 random assignment, 2000 re-samples) with the original (optimised) RF parameters for each model remaining consistent. For density plots (i), solid vertical lines denote the median AUC-ROC, and dashed lines denote the high and low 95% confidence intervals, for each response. Box-and-whisker plots (ii) show the AUC-ROC values for each re-sample; Matched re-sample permutations are connected by grey lines (i.e. same random assignment). The matched re-sample AUC-ROC values were significantly different between all groups (Wilcoxon SRT p value < 0.00001). (**B**) Density plots and box-and-whisker plots for 1000 re-samples with the training/validation set randomly assigned in 60/40 (i & ii) and 70/30 (iii & iv) ratios, respectively. (**C**) RF model re-sample (n=1000) prediction AUC-ROC with training/validation set assignment of 60/40 (i) and 70/30 (ii) ratios; plots show median (solid lines) and 95% CIs (dashed lines). (**D**) Density plot of the LPS-induced IFN module gene network connectivity determined by a Spearman's correlation matrix. Plot is stratified by individuals who did (orange) and did not (grey) record an sLRI in the first year of life. Related to Figure 4C(i). (**E**) Wiring diagrams and top drivers of the CBMC Imiquimod-induced IFN module stratified by individuals who were resistant (i) and susceptible (ii) to sLRIs in the first year of life. Wiring diagram and bar plot characteristics are the same as Figures 3B and 3C, respectively. (iii) Density plot of the Imiquimod-induced IFN module gene network connectivity stratified by individuals who did (orange) and did not (grey) record an sLRI in the first year of life. Related to Figure 4C-E. (**F**) Same analysis as above (Figure S6E) for the Poly(I:C)-induced IFN module. (**G**) Scatter plot of ranked gene expression and connectivity of the CBMC LPS-induce IFN module genes of individuals resistant (i) and susceptible (ii) to sLRIs in infancy (x-axis), compared to all 5yr PBMC samples (y-axis). (**H**) Box plots of the CBMC LPS-induce IFN module connectivity between individuals resistant (grey, left) and susceptible (orange, right) to sLRIs in infancy compared to 5yr PMC samples (magenta). Plots show median, 25th and 75th quartiles, and ±1.5xIQR; p values determined by Mann-Whitney U test.

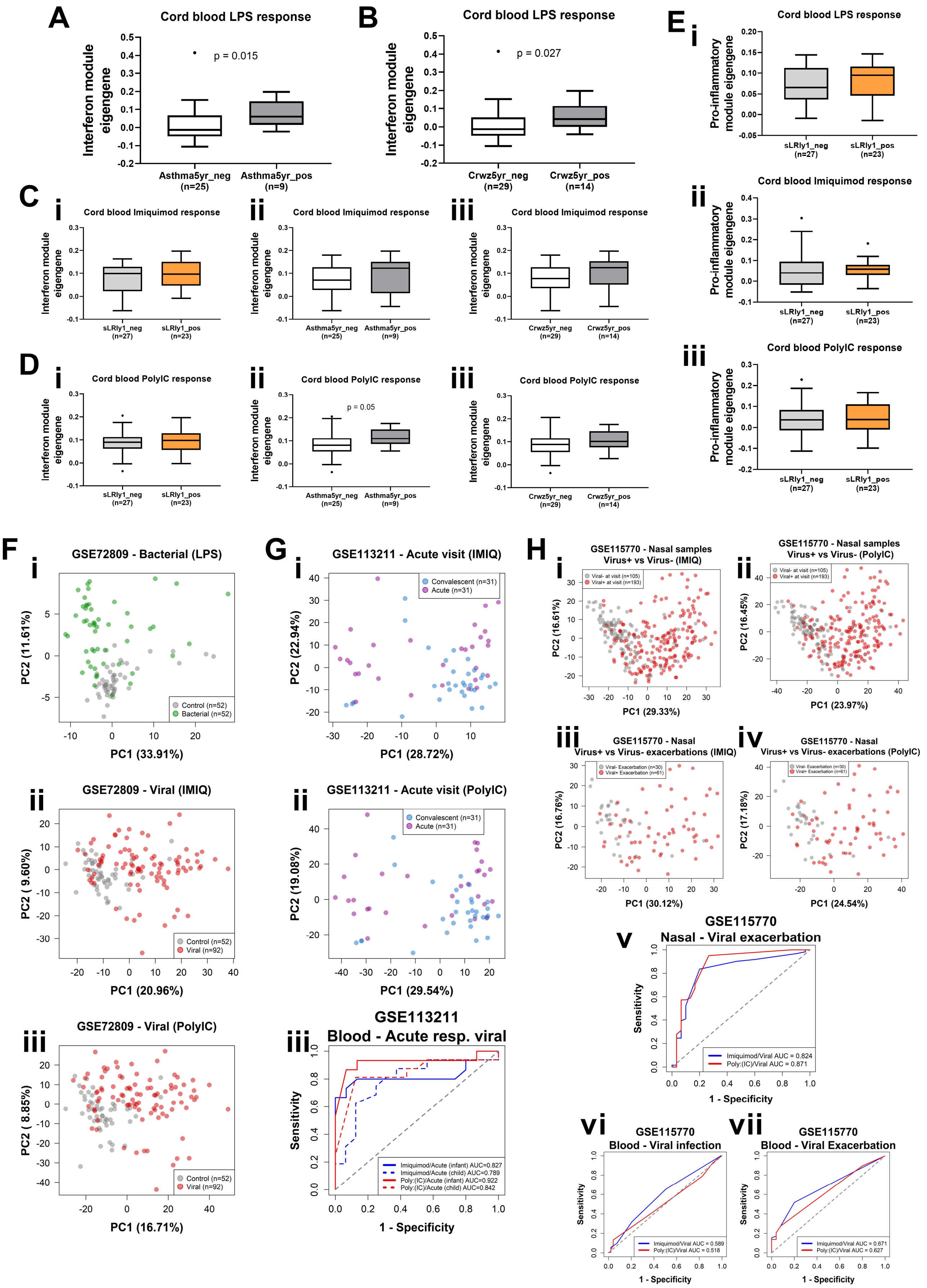
A
Cord blood LPS response
Interferon module eigengene
p = 0.015
Asthma5yr_neg (n=25)
Asthma5yr_pos (n=9)
B
Cord blood LPS response
Interferon module eigengene
p = 0.027
Crwz5yr_neg (n=29)
Crwz5yr_pos (n=14)
E i
Cord blood LPS response
Pro-inflammatory module eigengene
sLRly1_neg (n=27)
sLRly1_pos (n=23)
C i
Cord blood Imiquimod response
Interferon module eigengene
sLRly1_neg (n=27)
sLRly1_pos (n=23)
ii
Cord blood Imiquimod response
Interferon module eigengene
Asthma5yr_neg (n=25)
Asthma5yr_pos (n=9)
iii
Cord blood Imiquimod response
Interferon module eigengene
Crwz5yr_neg (n=29)
Crwz5yr_pos (n=14)
ii
Cord blood Imiquimod response
Pro-inflammatory module eigengene
sLRly1_neg (n=27)
sLRly1_pos (n=23)
D i
Cord blood PolyIC response
Interferon module eigengene
sLRly1_neg (n=27)
sLRly1_pos (n=23)
ii
Cord blood PolyIC response
p = 0.05
Interferon module eigengene
Asthma5yr_neg (n=25)
Asthma5yr_pos (n=9)
iii
Cord blood PolyIC response
Interferon module eigengene
Crwz5yr_neg (n=29)
Crwz5yr_pos (n=14)
iii
Cord blood PolyIC response
Pro-inflammatory module eigengene
sLRly1_neg (n=27)
sLRly1_pos (n=23)
F i
GSE72809 - Bacterial (LPS)
PC2 (11.61%)
PC1 (33.91%)
Control (n=52)
Bacterial (n=52)
ii
GSE72809 - Viral (IMIQ)
PC2 (9.60%)
PC1 (20.96%)
Control (n=52)
Viral (n=92)
iii
GSE72809 - Viral (PolyIC)
PC2 (8.85%)
PC1 (16.71%)
Control (n=52)
Viral (n=92)
G i
GSE113211 - Acute visit (IMIQ)
Convalescent (n=31)
Acute (n=31)
PC2 (22.94%)
PC1 (28.72%)
ii
GSE113211 - Acute visit (PolyIC)
Convalescent (n=31)
Acute (n=31)
PC2 (19.08%)
PC1 (29.54%)
iii
GSE113211
Blood - Acute resp. viral
Sensitivity
1 - Specificity
Imiquimod/Acute (infant) AUC=0.827
Imiquimod/Acute (child) AUC=0.789
Poly:(IC)/Acute (infant) AUC=0.922
Poly:(IC)/Acute (child) AUC=0.842
H i
GSE115770 - Nasal samples
Virus+ vs Virus- (IMIQ)
Viral- at visit (n=105)
Viral+ at visit (n=193)
PC2 (16.61%)
PC1 (29.33%)
ii
GSE115770 - Nasal samples
Virus+ vs Virus- (PolyIC)
Viral- at visit (n=105)
Viral+ at visit (n=193)
PC2 (16.45%)
PC1 (23.97%)
iii
GSE115770 - Nasal
Virus+ vs Virus- exacerbations (IMIQ)
Viral- Exacerbation (n=30)
Viral+ Exacerbation (n=61)
PC2 (16.76%)
PC1 (30.12%)
iv
GSE115770 - Nasal
Virus+ vs Virus- exacerbations (PolyIC)
Viral- Exacerbation (n=30)
Viral+ Exacerbation (n=61)
PC2 (17.18%)
PC1 (24.54%)
v
GSE115770
Nasal - Viral exacerbation
Sensitivity
1 - Specificity
Imiquimod/Viral AUC = 0.824
Poly:(IC)/Viral AUC = 0.871
vi
GSE115770
Blood - Viral infection
Sensitivity
1 - Specificity
Imiquimod/Viral AUC = 0.589
Poly:(IC)/Viral AUC = 0.518
vii
GSE115770
Blood - Viral Exacerbation
Sensitivity
1 - Specificity
Imiquimod/Viral AUC = 0.671
Poly:(IC)/Viral AUC = 0.627

Figure S7. Only the LPS-induced IFN is predictive of sLRI susceptibility in infancy and CBMC gene expression profiles are relevant in real world contexts. (**A**) Box-and-whisker plot of the cord blood LPS-induced IFN module eigengene, grouped by individuals who are asthmatic/non-asthmatics at 5 years of age. (**B**) Box-and-whisker plot of the cord blood LPS-induced IFN module eigengene, grouped by individuals who did or did not have wheeze in the 5th year of life. (**C**) Box-and-whisker plots stratified by sLRI susceptibility in infancy (i), asthma at 5 years (ii), and current wheeze at 5 years (iii) of the Imiquimod-induced IFN module eigengene. (**D**) Same analysis as above (Figure S7C) for the Poly(I:C)-induced IFN module eigengene. (**E**) Box-and-whisker plots of the proinflammatory module eigengene stratified by sLRI susceptibility in infancy of the LPS- (i), Imiquimod- (ii), and Poly(I:C)- (iii) response. This panel runs from top to bottom. All box-and-whisker plots above (Figure S7A-E) are related to Figure 4 and show median, 25th and 75th quartiles and whiskers are determined by the Tukey method. (**F**) Principal component analysis (PCA) of blood-derived gene expression profiles of children hospitalized with febrile bacterial and viral infections (GSE72809). Gene expression data sets were restricted to available CBMC LPS-induced (i), Imiquimod-induced (ii), and Poly(I:C)-induced (iii) IFN module genes, respectively. x and y axes show scores for the 1st and 2nd principle components and the proportion of variation attributed to each component, respectively. Relates to Figure 5A. (**G**) PCA of PBMC-derived gene expression profiles of infants and children hospitalised with acute viral (RSV) bronchiolitis (GSE113211). Gene expression data sets were restricted to available CBMC Imiquimod-induced (i) and Poly(I:C)-induced (ii) IFN module genes, respectively. Area under the ROC curve plot (iii) showing RF model prediction accuracy for samples collected from infants (solid lines) and children (dashed lines) separately. Relate to Figure 5B. (**H**) PCA of nasal-derived gene expression profiles of asthmatic children with viral+ and viral- cold-like illness (GSE115770). Gene expression data sets were restricted to available Imiquimod-induced (i) and Poly(I:C)-induced (ii) IFN module genes, respectively. Nasal-derived gene expression profiles of asthmatic children were filtered to only those who subsequently experienced an exacerbation; PCA of this subset with data sets restricted to available Imiquimod-induced (iii) and Poly(I:C)-induced (iv) IFN module genes, respectively. Relates to Figure 5C.

Supplementary methods

Study cohort

The study population consisted of a subset of 50 individuals from the Childhood Asthma Study (CAS), a prospective birth cohort at high risk for asthma development. This study was conducted between 1996-99 in Perth, Western Australia, and followed-up over the first 10 years of life. 263 infants were enrolled prenatally from expecting parents where at least one parent had a doctor-diagnosed history of asthma, hay fever or eczema. 235 (89.35%) and 198 (75.3%) study participants remained at the 1 and 5 year follow-ups, respectively. Fully informed written consent was obtained from the parents before recruitment, and approval for the study was obtained from the relevant local ethics committees. Relevant clinical and home environment information was collected from the parents, and birth information was collected post-delivery. All respiratory illnesses were closely followed-up through the use of daily symptom diaries during episodes of acute respiratory illnesses and fortnightly telephone calls until resolution of symptoms. Recorded symptoms included runny/blocked nose, cough, wheeze, and fever. Upper respiratory infections (URI) were defined as any episode of runny/blocked nose or cough which did not present with difficulty breathing/tachypnea, wheeze, or a rattly chest. Lower respiratory infections (LRI) were considered mild if they involved only a rattly chest and were considered severe (sLRI) if they involved a rattly chest in addition to wheeze and/or fever. Rattle (rattly chest) was defined as wet noisy breath sounds heard from the child's chest, whereas wheeze was defined as audible, expiratory, high-pitched whistling sounds. Fever was defined by recording a temperature >38°C (digital thermometer) on two occasions measured more than 1 hour apart, within 48 hours of the onset of respiratory infection symptoms. The study centre was contacted within 24 hours of the onset of respiratory infection symptoms and a home visit was arranged (within 48 hours) to collect nasopharyngeal aspirate (NPA) samples, which were stored at -80°C. NPAs were also collected during predetermined periods when infants/children were well and had not experienced respiratory symptoms for ≥4 weeks at 2, 6, and 12 months of age, then half-yearly (winter/summer) to 5 years. NPAs were analysed by RT-PCR for detection of human Rhinoviruses, Respiratory Syncytial virus, influenza A and B, coronaviruses 229E and OC43, parainfluenza viruses 1–3, adenoviruses, and Human Metapneumovirus. Clinical assessments were conducted periodically over the study period to determine relative outcomes. Current wheeze at 5 years (Crwz5) was defined as any wheezy event recorded (parental assessment) in the 12 months before the 5 year follow-up. Asthma at 5 years was defined as having doctor diagnosis of asthma ever, a prescription to asthma medication, and current wheeze at 5 years. A non-asthmatic determination at 5 years had none of these criteria. Umbilical cord blood was collected at birth and peripheral blood was collected at 0.5, 1, 2, 3, 4, 5, and 10 years (as

close to the birth date as possible). Replicate samples were processed and cryopreserved within approximately 8 to 12 hours of collection, as previously described(2). Atopy was determined by skin prick tests (SPT) to a panel of seven allergens (house dust mite, cat dander, ryegrass, *Alternaria*, *Aspergillus*, cow's milk, and egg white) at 6 months, and 2, 5 and 10 yrs. Positive (Histamine) and negative (saline) controls were included. Wheal size ≥2 mm (60mo, 2yr) or ≥3 mm (5/10yrs) after 15 minutes was consider atopic (allergic). The CAS cohort has previously been characterised in detail(3-9).

Flow cytometry

Cryopreserved CBMCs were thawed and washed with RPMI 1640 (Gibco) containing 10% non–heat-inactivated FBS (Serana Australia). 10µl of cell mixture was stained with trypan blue and counted with a haematocytometer. Approximately $1x10^6$ cells were aliquoted for immunophenotyping, and $0.25x10^6$ cells were aliquoted for unstained controls, for each sample. Cells were pelleted by centrifugation at 1500rpm (~500g) for 5 minutes at 4°C, and excess media was removed by vacuum aspiration. Each sample was incubated with 50µl of a master mix of monoclonal antibodies (CD19-FITC *RRID: AB_395812*, CD3-AF700 *RRID: AB_396952*, CD4-V500 *RRID: AB_1937323*, CD14-APC-Cy7 *RRID: AB_1645464*, HLA-DR-PerCP-Cy5.5 cat #347364, CD25-BV421 *RRID: AB_11154578*, CD127-BV605 *RRID: AB_2738138*, CD123-CF594 *RRID: AB_11153664*, CD11c-PE-Cy7 *RRID: AB_10611859* [BD Bioscience] and FcεRIα-APC *RRID: AB_10671394* [eBioscience] (intracellular FoxP3 antibody added separately, below)) in cold FACS Buffer (PBS + 1% BSA) for 30 minutes at 4°C in the dark. Cells were washed, fixed and permeabilized with (Cytofix/Cytoperm buffer (BD Biosciences)) for 1 hour, and incubated for 30 minutes with FoxP3-PE *RRID: AB_10563418* (intra-cellular, BD Biosciences). The same antibody batches were used for all samples at the manufacturers recommended dilution. Individual cells were acquired using the LSR-Fortessa platform with FACSDiva software (BD Biosciences) following quality control assessment (Rainbow calibration and CS&T beads (BD Biosciences) prior to each cytometry run and unstained control were included for each sample. Initially, samples were compensated and gated with FlowJo 10.3 software. Compensated FCS files were imported into the R (3.6.2) statistical environment and pre-processed with the *flowWorkspace* and *flowCore* packages. Logicle transformation (*flowCore)* and batch correction (*sva*) was applied to all samples. Nonparametric paired (Wilcoxon signed rank test) or unpaired (Mann-Whitney U test) tests were used to determine between group differences and cell composition was assessed by dimensionality reduction (UMAP, *uwot* R package).

In vitro cell culture

CBMCs available for analysis were randomized with sLRI incidence in the first year of life. When available, matched 5 year PBMC samples were cultured alongside their CBMC counterpart (same batch, block randomization), so that analysis between stimuli and age accounted for potential cell culture batch effects. Cryopreserved samples were removed from liquid nitrogen storage and thawed in RPMI 1640 (Gibco) + 10µl DNase. Samples were washed (centrifuge (1340rpm, ~ 400g) for 7 mins at RT) and resuspended in 1ml PBS + 2% AB serum. Red blood cells were depleted from cord blood samples with an EasySep kit and "easy eight" magnetic separation mount (Stemcell Technologies), as per the manufacturers recommendation. 5 year PBMC samples were treated identically, without the depletion reagent. 10µl of each sample was stained with trypan blue and counted with a haematocytometer. Cells were resuspended at $1x10^6$ per ml in RPMI + 5% AB serum. $0.25x10^6$ cells (250µl) were transferred to dedicated wells of round bottom 96-well polystyrene culture plates (ThermoFisher Scientific). Wells were stimulated with LPS (Enzo Biochem, Cat No. ALX-581-007-L001, 1ng/ml) (derived from E. *coli*, serotype R515), Imiquimod (Invivogen, Cat. Code: tlrl-imq, 5µl/ml) or Poly(I:C) (Invivogen, Cat. Code: tlrl-pic, (50µl/ml), or left untreated. Stimuli was also added to 250µl RPMI + 5% AB serum (no biological material) for metabolite QC. Culture plates were incubated for 18 hours at 37^oC (5% CO_2). Immediately post-culture, supernatants from 4 wells ($1x10^6$) were pooled, mixed, aliquoted for back-up samples, and snap frozen by immersion in liquid nitrogen for metabolomics analysis. Supernatant for cytokine profiling was collected and stored at -20^oC. Cell pellets from 4 wells (~$1x10^6$ cells) were washed with RPMI + 5% AB serum, pooled, and stored at -20^oC in Trizol (Invitrogen).

Data generation

RNA-Seq: RNA was extracted with RNeasy MinElute Kits (Qiagen) in batches and the extraction batch information was recorded. RNA concentration was measured (Bioanalyzer; Agilent, Santa Clara, USA) and found to be good quality (RIN score; mean = 8.514, 95%CI = 8.46-8.567). A low yield protocol was employed with sequencing libraries prepared with NEBNext Ultra II Kits (New England BioLabs, Massachusetts, USA) and sequenced with the NovaSeq 6000 (Illumina, San Diego, USA) platform at the Australian Genome Research Facility (AGRF, Melbourne, Australia) for sequencing (100bp paired-end).

Cytokines: The concentrations of 48 cytokines were simultaneously quantified using the Bio-Plex Pro Human Cytokine Screening Panel (BioRad) with the Luminex 100/200 platform (Luminex). Analyte quantification (pg/ml) was determined by alignment to a standard curve. The cytokine panel included

CTACK, FGF basic, Eotaxin, G-CSF, GM-CSF, GRO-α, *HGF*, IFN-α2, IFN-γ, IL-1β, IL-1ra, IL-1α, IL-2, IL-2Rα, IL-3, IL-4, IL-5, IL-6, IL-7, *IL-8*, IL-9, IL-10, IL-12(p40), IL-12(p70), IL-13, IL-15, IL-16, IL-17A, *IL-18*, IP-10, LIF, MCP-3, MCP-1, M-CSF, MIF, MIG, MIP-1α, MIP-1β, β-NGF, *PDGF-BB*, SCF, *SCGF-β*, SDF-1α, RANTES, TNF, TRAIL, and VEGF.

Metabolites: Untargeted metabolomics data were acquired using an Ultimate 3000 UHPLC system coupled to a Thermo Q-Exactive Focus (Orbitrap) mass spectrometer (Thermo Fisher Scientific, Waltham, USA). Two chromatographic methods were used: reversed phase (C18) and hydrophilic interaction chromatography (HILIC). Data were acquired across 3 sequential analytical batches per mode, with 17 pooled QCs being analysed per batch (two pooled QCs at the beginning of each batch, then one after every 8th experimental sample). On the day of acquisition, 50µL of cold LC-MS grade methanol (C18) or acetonitrile (HILIC) containing internal standards were added to 50µL of cell culture supernatant. Samples were mixed at 1400 rpm and 4°C for 60 seconds, then centrifuged at 12,000 rpm and 4°C for 10 minutes to pellet the protein. 50µL of supernatant were transferred to an LC-MS vial before being placed in an autosampler at 6°C for analysis. For C18 metabolomics, the metabolites were separated with a Hypersil Gold column (100 x 2.1mm, 1.9µm particle size; Thermo Fisher Scientific, Waltham, USA). The mobile phase solvents were water containing 0.1% formic acid (solvent A) and acetonitrile containing 0.1% formic acid (solvent B). The elution gradient was as follows: initial conditions 1% [B], increasing to 50% [B] in 2 minutes, 50 to 99 % [B] in 7 minutes, held at 99% [B] for 2 minutes, return to initial conditions in 30 seconds, and equilibrating at initial conditions for 3.5 minutes. The flow rate was 0.3 mL/min and the column temperature was 45°C. For HILIC metabolomics, the metabolites were separated with an Acquity BEH Amide column (100 x 2.1mm, 1.7µm particle size; Water Corp, Milford, USA). The mobile phase solvents were water containing 10 mM ammonium formate and 50 mM formic acid (solvent A, pH=3) and 90% acetonitrile containing 10 mM ammonium formate and 50 mM formic acid (solvent B). The elution gradient was as follows: 1 minute at 100% [B], 100% to 60% [B] in 7 minutes, 60% [B] for 1 minute, then return to initial conditions (100% [B]) in 1 minute before equilibrating at initial conditions for 5 minutes. The flow rate was 0.4 mL/min and the column temperature was 35°C. For both modes, 6 µL of sample were injected in the column. Data were acquired in a mass range of 70 to 1000m/z using the following settings: positive electrospray ionisation (ESI); spray voltage, 3.5kV, sheath gas, 50 arbitrary units (au), auxiliary gas, 10au; and capillary temperature, 320°C (C18) and 350°C (HILIC). Data were acquired in full scan with a resolving power of 70,000 full width half maximum (FWHM) and data dependent MS fragmentation (ddMS) with a resolving power of 17,500 and at 20eV.

Data pre-processing

RNAseq: The binary base call (BCL) sequence files were converted to fastq files with the bcl2fastq pipeline (Illumina). Sequence data were processed with MEdical Sequence Analysis Pipeline (MESAP) (available at https://github.com/kim-carter/mesap) and aligned to the hg38 genome with HISAT2(10) and counts were quantified with *summariseOverlaps* function from the GenomicAlignments R package, with mode set to 'Union'. Pre- and post-alignment QC was assessed with FastQC (http://www.bioinformatics.babraham.ac.uk/projects/fastqc), MultiQC(11) and SAMStat(12). All samples were assigned 'PASS' (and not 'WARN' or 'FAIL') from FastQC for the categories 'Basic statistics', 'Per base quality', and 'Per sequence quality'. The average percentage of mapped reads was 95.06%% (range 87% to 96.9%) and the average total sequences per sample (millions) was 38.55 (range 10.7% to 56.8).

Cytokines: Cytokines were excluded if more than 30% of samples (excluding unstimulated samples) recorded out of range (OOR) values. This resulted in the removal of CTACK, IL-3, IL-7, IL-8, IL-13, IL-18, PDGF-BB, SCGFβ, SDF-1α from further analysis. Remaining OOR values were imputed below/above the minimum/maximum based on truncated normal distribution with the *rtruncnorm* function from the truncnorm R package, so that partial information is used to define values below/above the limit of detection for imputation. The most appropriate method of transformation and normalisation was tested (data not shown), and ArcSinh transformation and Loess normalisation was applied. Batch effects were removed with linear modelling (*removeBatchEffect* function from the limma R package).

Metabolites: Raw spectral files were processed using Compound Discoverer v3.0 (Thermo Fisher Scientific, Waltham, USA), yielding relative abundances of metabolites. Metabolite annotations and identifications were made by screening against an in-house spectral and retention time metabolite library and a vendor-specific spectral database (www.mzCloud.org). To account for adduct ions and fragmentation ions not detected in Compound Discoverer, clustering of similar features was performed as described(13). Analytical drift was corrected for using robust spline correction(14). Metabolites were retained for further analysis if they fulfilled the following criteria: (1) a molecular weight match (±0.0001 Daltons), (2) a mass error < 5 ppm, (3) an MS^2 (MS/MS fragmentation) spectral match to either an in-house spectral library or the mzCloud database, (4) a relative standard deviation (RSD) of the QC samples < 20%(15), and (5) an identifier in the Human Metabolome Database (www.hmdb.ca). Where possible, chromatographic retention time matches were also made against an in-house library. This strategy ensured only metabolites with a high quality and annotation confidence were included for downstream analysis. This process identified one sample as an outlier, which was removed; A

Poly(I:C)-stimulated cord blood sample with corresponding samples at 5 years. Due to the experimental design, the corresponding samples of the outlier were also removed when paired analysis was required.

Dimensionality reduction

The experimental design ensured that matched data was generated from 4 conditions (Unstimulated, LPS-, Imiquimod-, and Poly(I:C)-stimulated) for each individual within the same batch. This allows for a multi-level design for dimensionality reduction analysis (Principal Component Analysis and Canonical Variate Analysis), whereby the within subject variance is decomposed from the between subject variance, which considerably improves the power and interpretability of subsequent multivariate analysis(16, 17). For this purpose, the *withinVariation* function was adapted from the mixOmics package in R(18).

Transcripts: Following pre-processing, CBMC gene expression data for matched Unstimulated, LPS-, Imiquimod-, and Poly(I:C)-stimulated samples for the 50 individuals was filtered to reduce noise, to only significantly variable genes, with the *varianceBasedfilter* function in R. For this analysis, genes were considered significant with a p value lower than a strict threshold of $2.88x10^{-6}$, determined by 0.05/number of genes (n=17356). This resulted in 5,885 genes for dimensionality reduction. Within subject variation was calculated with the *withinVariation* function, genes were scaled to unit variance and the *PCA* function from the FactoMineR package was used for principal component analysis(19) and the principle component scores and variable contributions were used for plots in Figure 1.

Cytokines: Following pre-processing, within subject variation was calculated (as above) from the CBMC cytokine concentration data (n=39) for matched Unstimulated, LPS-, Imiquimod-, and Poly(I:C)-stimulated samples for the 50 individuals. Principal component analysis was applied as described above.

Metabolites: Following pre-processing, within subject variation was calculated (as above) from the CBMC metabolite relative abundances (n=49) for matched Unstimulated, LPS-, Imiquimod-, and Poly(I:C)-stimulated samples for the 49 individuals. The data was scaled to unit variance with the base R *scale* function. The *CVA* function from the Morpho package was used for canonical variate analysis with Jack knife cross-validation with 2000 permutations. The cross-validated canonical variate scores were used for plots in Figure 1 and Supplementary figure 2.

Transcriptomics analysis

Differential expression analysis.

EdgeR(20)

A total of 50,019 raw transcripts were available for analysis following pre-processing. Raw transcripts were removed if they had no counts in any sample, lacked annotation, or had 0.5 counts per million in ≤ 25 samples. This strategy produced 17,363 transcripts for analysis. Data was normalised with the trimmed mean of M-values (TMM normalisation(21)). As the experimental design involved block randomisation (with respect to age and stimuli) into batches, the was no batch effect related to cell culture batch number for these paired comparisons. There was no discernible batch effect observed for unpaired comparisons, however culture batch was included as covariate for unpaired analysis (i.e. sLRI susceptibility in infancy) regardless. Unwanted variation was identified and removed with the *RUVg* function from the RUVSeq R package(22), which models a set of empirical control genes (not significantly different between any comparison of interest) to determine putative technical effects (lane, sequencing, etc). A paired designed was employed for analysis between matched stimulated and unstimulated samples, which included trends identified by *RUVg* as covariates. The EdgeR pipeline was run with default parameters, which includes the *estimateDisp*, *glmQLFit*, *glmLRT*, and *topTags*, which fits a negative binomial generalized log-linear model to the counts for each gene and conducts genewise likelihood ratio tests. For analysis between CBMC and matched samples at age 5 years (n=27), a paired design was employed which modelled differences between matched unstimulated and the corresponding simulated samples, as well as RUVg trends. An unpaired design which modelled unstimulated/stimuli and RUVg trends was used to determine differences between the primary outcome. Genes were considered significantly different if it recorded an FDR-adjusted Benjamini-Hochberg p value < 0.01 and a Log_2 fold change above 1 (upregulated) or below -1 (downregulated). A matrix of corrected gene counts was generated for downstream network analysis. Size factors were estimated using the median ratio method with the *estimateSizeFactors* function and variance stabilizing transformation (VST) was applied to the count data with the *varianceStabilizingTransformation* function, from the DESeq2 package(23). RUVg trends which related to technical variation and culture batch were removed as covariates with a linear model with the *removeBatchEffect* function from the limma package.

Limma-voom(24)

Transcript filter, normalization and model design was performed the same as described for EdgeR analysis. The data was transformed to $\log_2$-counts per million and the mean-variance relationship was estimated to produce weights using the *voom* function. The limma pipeline was run with default parameters, which includes the *lmFit*, *contrasts.fit*, *eBayes*, and *topTable* functions. The same criteria as the EdgeR analysis was applied to determine gene significance. From this analysis, the moderated t-statistic calculated for each gene was plotted by module for each network, as a way of displaying which modules are differentially regulated between simulated and matched unstimulated samples. The moderated t-statistic is the ratio of the M-value ($\log_2$-fold change) to its standard error, which has been "moderated" (empirical Bayes) across all genes. Applying the module eigengene instead of the moderated t-statistic yielded the same overall result with respect to module up-/down-regulation (data not shown). Modules with medians above a moderated t-statistic of 2 are considered significantly upregulated and those below -2 are considered significantly downregulated.

Weighted Gene Co-expression Network Analysis (WGCNA(25, 26))

Corrected count data (described above) were used as input which included 17,363 genes for analysis. For this analysis, three perturbation networks were created, each of which included unstimulated samples and the corresponding stimulated samples (i.e. the LPS, Imiquimod, and Poly(I:C) networks). The varianceBasedfilter function was used to filter significantly variable genes (p value < 0.01) for each condition, and union genes between the unstimulated and respective stimulated samples. This strategy resulted in 6561, 6757, and 6764 genes available for the LPS, Imiquimod, and Poly(I:C) networks, respectively. Soft powers were calculated with the *pickSoftThreshold* function with the networkType parameter set to "signed". This resulted in soft powers of 7, 8, and 7 for the LPS, Imiquimod, and Poly(I:C) networks, respectively (Figure S3, above). Adjacency and topological overlap matrices (TOM) were created with the *adjacency* and *TOMsimilarity* functions, respectively, with "signed" network specified. The TOM dissimilarity matrices were calculated by one minus the TOM similarity matrix (1-TOM). Modules were identified by hierarchical clustering with the *hclust* function (method = "average") and pruned with the *cutreeDynamic* function (method = "hybrid", deepSplit = 2, minClusterSize = 50). TOM plots (Figure S3) were created with the *TOMplot* function. Module eigengenes were calculated with the *moduleEigengenes* function. Modules were merged if they were similar, determined by correlation of their eigengenes, hierarchical clustering, and dendrogram cut at 0.1 with the *mergeCloseModules* function. Network statistic and within module connectivity was calculated with the *intramodularConnectivity* function. Modules were annotated with a consensus approach by assessing

significantly enriched pathways from: Gene Ontology term enrichment (*GOenrichmentAnalysis*), ReactomePA(27) and clusterProfiler(28) R packages, InnateDB(29), and identification of top module genes (Log_2-FC/gene connectivity). Additionally, we employed a separate analysis to confirm that the principal modules of interest related to innate immune function we identified following WGCNA analysis (i.e. the interferon and proinflammatory modules) were captured within the blood transcriptional module repertoire from BloodGen3Module(1). The BloodGen3 module repertoire consists of 382 individual modules (gene sets) defined from 16 reference cohorts (985 unique transcriptome profiles) representing a range of disease settings(1, 30). For this analysis, input genes were filtered firstly to those which had >5 transcript counts in at least 20 samples, and secondly to only those genes which were represented in at least one module from either the LPS-, Imiquimod-, and Poly(I:C)-based networks. This resulted in 6,562 genes available for comparison with the BloodGen3 module repertoire. 2 genes ("LINC01505" "SDHAP2") were represented in a module but did not pass the count-base filter and were excluded. Gene counts were normalized with respect to sequence depth with the *SCnorm* function from the SCnorm R package with default parameters(31). The data was then filtered to stimulated/Control genes for CBMC samples, and the *Groupcomparison* function from the BloodGen3Module R package was used to perform group comparison (t-test) between stimulated and unstimulated samples at the module level. Annotated fingerprint grid plots were produced with the *gridplot* function from BloodGen3Module, which displays each module in a fixed position, where red (blue) dots represent modules in which member genes are predominantly increased (decrease) from the group-wise comparison. Rows are organised in "Aggregates" of modules which are considered functionally-related module sets. In general, there was consistent consensus of biological function across annotation/enrichment methods. This analysis was run with R version 4.1.1. Module preservation between networks was calculated with the *modulePreservation* function, with 200 permutations, networkType set to "signed", and the "gold" (random) module size set to the average module size for each comparison. The ranked expression was calculated as the (rank) average expression of each genes across all samples, and ranked connectivity was calculated with the (rank) softConnectivity function, with type set to "signed" and power set to the corresponding network's soft power. Soft connectivity is defined as the sum of the adjacency (co-expression measure) of each gene in the network to all other genes. Connectivity density were determined with the *density* function and the Sheather-Jones smoothing bandwidth method was used. Connectivity densities were assessed for normal distribution with a Lilliefors test of normality (*lillie.test* function). A Spearman's Rho correlation matrix was also calculated for each module to separately assess intramodule connectivity, defined as the sum of the correlation value of each gene to all other genes. Network wiring diagrams of the top 20

most connected genes were constructed with the *graph_from_adjacency_matrix* function from the igraph R package. Node size represents number of connections (degree) among the total network and edge with indicates strength of connection (red edges denote a correlation > 0.8).

Master regulator analysis

Master regulators were identified by employing the Algorithm for the Reconstruction of Accurate Cellular Networks-Adaptive Partitioning (ARACNe-AP)(32, 33), Virtual Inference of Protein-activity by Enriched Regulon analysis (VIPER)(34) and RcisTarget(35). First, a reverse engineered regulatory network was constructed with ARACNe-AP, which employs Adaptive Partitioning (AP) for estimating the Mutual Information. The algorithm was run as a java executable with 100 bootstraps and a mutual information threshold of ~ 0.092. Input data consisted of the corrected count data for all samples and all genes (n=17,363) and a list of transcription factors. Transcription factors used as input had the following criteria: classified as either “known motif”, “Inferred motif”, or “Likely to be sequence specific TF” by Lambert *et al.* 2018(36) and were present in the input data matrix. This strategy resulted in 1,224 transcription factor identifiers as input. Secondly, the ARACNe-AP network and corrected count data was inputted into the *aracne2regulon* function from the viper R package to create a regulon object. The resulting regulon object and gene expression data from comparison groups were used to create a gene expression signature with the *bootstrapTtest* function (1000 permutations) and master regulator inference analysis was employed with the *msviper* function. The *ledge* function was used for leading edge analysis and the *bootstrapmsviper* function was used to integrate bootstrapping. Finally, putative master regulators were trimmed with RcisTarget, which selects DNA motifs that are significantly over-represented transcription start site (TSS) of a set of genes, annotates the motif to TF, filters on a normalized enrichment score, and predicts candidate target genes. The motif database was from the human hg38 genome assembly and covered the region 500bp upstream and 100bp downstream of TSS. Motifs were identified with the *calcAUC* and *addMotifAnnotation* functions. Ultimately, RcisTarget was employed as an orthogonal (database-driven) method to trim the (data-driven) results of ARACNe/VIPER, so that master regulators were only retained if their putative target genes were also enriched for DNA binding motifs in the region of TSSs of the same transcription factor.

CIBERSORTx(37)

To create reference profiles, scRNAseq profiles of 262,332 cord blood cells were downloaded from the Human Cell Atlas(38) as a .loom file (file name: 1M-immune-human-blood-10XV2.loom; available at https://data.humancellatlas.org/explore/projects/cc95ff89-2e68-4a08-a234-480eca21ce79, accessed on 07/04/2021). The cell annotations included were as follows:

Cell type	Number	Cell type	Number
CD14_monocyte	28352	naive_B_cell	36288
CD4_T_cell	2513	naive_CD8_T_cell	33485
dendritic_cell	1658	naive_T-helper_cell	69214
erythroid_cell	4820	naive_T_cell	59907
megakaryocyte	2506	natural_killer_cell	14553
memory_B_cell	1689	T-helper_cell_including_regulatory_T_cell	7347

The cells were randomly down-sampled to 1,000 cells per cell type to ensure even representation across cell types. Genes were filtered to the 5,000 most variable. The Seurat(39) R package (version 3) was used for pre-processing. The *PercentageFeatureSet* function was used to estimate the proportion of mitochondrial gene expression, the *NormalizeData* function was used to normalize the data ("LogNormalize", scale.factor = 10000), and the *SCTransform* function was used for transformation and to regress out mitochonidrial gene proportion. The *umap* function from the uwot R package was used for dimensionality reduction (Figure S2), with default parameters. The *vst* function from the sctransform R package was used for variance stabilising transformation of the dataset to be exported, with processing batch included as an interaction term. This approach was decided on to remain consistent with the bulk RNAseq profiles. CIBERSORTx was run on the bulk RNAseq mixture and scRNAseq reference profiles (non-log space) with default parameters and 100 permutations. The *addtable2plot* function from the plotrix R package was used to create results tables from between group comparisons.

Random forest models

For RF analysis in this study we used the gene expression data from gene modules defined by WGCNA, which by design clusters genes according to co-expression, so that module member genes exhibit high multicollinearity, which is a recognised influence on RF interpretation(40, 41). However, collinearity primarily affects the interpretation of variable importance, and not the overall model prediction accuracy. For this reason, the RF classifiers used in the present study were employed principally to test the utility of IFN module genes to predict outcomes, and the variable importance measures, should be considered an underestimation of their true value.

CAS cohort: To account for potential differences in baseline/unstimulated CBMC gene expression between individuals, ∆ values were taken from the matched stimuli gene expression profiles (e.g. LPS-stimulated gene expression – matched unstimulated gene expression = adjusted LPS-stimulated gene expression matrix), and these were used as input. Genes were filtered to only those present in the IFN modules of the corresponding response. Subjects (n=50) were randomly assigned to either a test or validation set (50/50 split), and the same random assignment was applied to the LPS, Imiquimod and

Poly(I:C) datasets. The *randomForest* function (randomForest R package) was to optimise each RF model, with respect to the number of variables randomly sampled as candidates at each split ("mtry") and the number of decision trees to grow ("ntree"), to classify individuals who did and did not experience an sLRI in infancy. For the mtry parameter, a sequence from the lower of 10 or the square root of the number of input genes up to five times the square root of the number of input genes, by an increase of one, was defined. RF classifiers were built for all numbers in the sequence, with ntree set to 1000, and the mtry value which recorded the lowest out-of-bag error rate (OOBer) was selected as optimal. The lowest number was selected in the case of a tie in OOBer. For the ntree parameter, a sequence from 500 to 10,000 increasing in increments of 100 was defined, and RF classifiers were built for all numbers in the sequence, with mtry set to the optimal as defined above. The optimal ntree was selected that produced the smallest OOBer (lowest number if a tie). This approach resulted in a mtry of 11, 23, and 121 and a ntree value of 5400, 1000, and 1000 for the LPS, Imiquimod, and Poly(I:C) RF models, respectively. The trained models, which internally bootstrap the training set (70/30 split) reported OOBer of 36%, 72%, and 52% for the LPS, Imiquimod, and Poly(I:C) RF models, respectively. Following optimisation, final RF classifiers were used to predict the primary outcome status in the corresponding validation set (without class labels) with the *predict* function (stats R package). The *prediction* and *performance* functions from the ROCR R package were used to compare the predictions to true values and to determine true and false positive rates to calculate and area under the Receiver Operating Characteristic (ROC) curve. The 20 genes which conferred the greatest decrease in model accuracy when not randomly selected (i.e. genes with the greatest importance) for each model were as follows: LPS-induced IFN model: KLHDC7B, IFNG, CASZ1, PSMB9, PARP3, ACOT7, NUB1, USP18, NLRC5, CCDC194, GCH1, PARP11, CXCL11, PMAIP1, IFIT5, GPR174, CD38, HERC5, SAMD9, OASL.

IMIQ-induced IFN model: KIR2DL3, RGS13, MAPK8IP2, MYO10, C1GALT1C1, WHAMMP3, HLA-K, ACP5, PMP22, NKAIN1, TEF, ATF5, NAPA, DTNB-AS1, WDFY1, TCF3P1, TNFSF10, HESX1, EIF2AK2, GLRX.

Poly(I:C)-induced IFN model: KCP, NUDT8, SPATA32, SUPT4H1, TUBB2BP1, OTUD7B, GK3P, LINC01871, PARP3, JUP, EPHB1, COL5A3, KCTD14, MDGA1, ZNF429, SLC31A2, NEFH, PRKG1, IL18RAP, NCOA4P2.

Training/validation set re-sampling: To test the reproducibility of the RF classifiers, the adjusted LPS, Imiquimod, and Poly(I:C) datasets were each randomly re-sampled 2000 times, with respect to their training/validation set assignment (50/50 split). The same 2000 random assignments were used for the LPS, Imiquimod, and Poly(I:C) classifiers. RF classifiers were built on the training set and tested on the

validation set (as above) for each re-sample (using the previously determined optimal parameters, above), and the area under the ROC curve was recorded each time to determine the prediction accuracy. To assess whether different proportional assignment of training and validation set produced similar results, RF classifiers of the adjusted LPS, Imiquimod, and Poly(I:C) datasets were each randomly re-sampled 1000 times (with respect to their training/validation set assignment) at training/validation assignments of 60%/40% and 70%/30%. The same 1000 random assignments were jused for the LPS, Imiquimod, and Poly(I:C) classifiers, and they were built with the above (optimised) parameters. The area under the ROC curve was recorded for each re-sample to determine the prediction accuracy.

External cohorts: We trained RF classifiers on our CBMC data and used them to classify samples derived from a series of publicly available data sets from the Gene Expression Omnibus. In general, we used unstimulated samples from the CAS cohort to represent “healthy” individuals (absence of infection), and stimulated samples to represent anti-bacterial (LPS) and anti-viral (Imiquimod/Poly(I:C)) innate immune responses of infants/children with confirmed infection. *GSE72809*: RF classifiers were trained on the adjusted LPS- and Imiquimod-/Poly(I:C)-stimulated CBMC gene expression datasets (n=50 each) and used to predict children hospitalised with bacterial (n=52) and viral (n=92) infections, respectively, from healthy controls (n=52), from blood-derived gene expression profiles. Model optimization was implemented and the model prediction accuracy was tested on the external gene expression profiles as previously described (above). *GSE113211*: RF classifiers were trained on the adjusted Imiquimod- and Poly(I:C)-stimulated CBMC gene expression datasets (n=50 each) and used to predict infants (≤ 18months, n=15) and young children (18mo-5yrs, n = 16) hospitalised with acute viral bronchiolitis from matched samples collected post-convalescence (symptom-free, 8.8 ± 2.5 weeks post-infection) from PBMC samples. Model optimization was applied and the model prediction accuracy was tested on the external gene expression profiles, as previously described (above), for all subjects together as well as infants and children separately. *GSE115770:* RF classifiers were trained on the adjusted Imiquimod- and Poly(I:C)-stimulated CBMC gene expression datasets (n=50 each) and used to predict study visits asthmatic children (6-17yrs) with viral-associated (n=193) “cold”-like illness from those with non-viral “cold”-like illness (n=105) (samples taken 1-6 days post-onset), some of which later experienced exacerbations (58 did and 25 did not). Model optimization was implemented and the model prediction accuracy was test as previously described (above), for viral infection given cold symptoms, and viral infection given exacerbation, for nasal- and blood-derived gene expression profiles separately.

DIABLO Integration

The Δ values (see above) of the transcriptomic, metabolomic, cytokine, and transcription factor (TF) activation (but not baseline immune cell proportion) datasets were calculated and used as input data for DIABLO integration from the mixOmics R package(42). The datasets were filtered to only LPS-stimulated CBMC samples. The transcriptomic data set was filtered to significantly variable genes (p<0.01, n=6344 genes), with the *varianceBasedfilter* function from the DCGL R package, to reduce noise from low variance gene expression (n=6344 genes). Importantly, no preference was given to genes separately identified in the LPS-induced IFN module. All other datasets were included in their entirety. Datasets were scaled to unit variance with the base R scale function. DIABLO requires a design matrix to indicate which datasets (blocks) should be connected to maximise covariance (correlation), and to what extent. For this analysis, a value of 0.125 was selected in the design matrix between each data set (diagonal set to 0) and was chosen as it approximates the difference between a null design matrix (maximise discrimination, all values set to 0) and the average principal component correlation between each dataset. The number of components and variables per block per component to use for the final model were optimised with the *perf* and *tune.block.splsda* functions (mixOmics), respectively. Due to the computational time required to optimise all variables per component simultaneously, multiple rounds of optimisation were run (5-fold cross-validation permutated 20 times) with selected numbers of variables per block to iteratively determine the final input number for each dataset (i.e. the keepX call of the *block.splsda* function (mixOmics)). The DIABLO model was run with default parameters with the *block.splsda* function of the mixOmic R package and the circos plot was created with the *circosPlot* function, displaying the 1st latent component.

Statistical analysis

All statistical analysis was computed in the R environment (version 3.6.2, unless otherwise stated) and graphs were produced from R or Prism software (version 8, GraphPad Software, La Jolla California USA). Non-parametric statistical methods were applied to test group differences (Mann-Whitney U test (unpaired analysis) and Wilcoxon signed Rank Test (paired analysis); *wilcox.test* function [stats R package]) and correlations (Spearman's rank correlation coefficient; *cor.test* function [stats R package]). For comparisons of study population characteristics (Table 1), Fisher’s Exact test (*fisher.test* function [stats R package]) was used to calculate odds ratios, 95% Confidence Intervals, and accompanying P values for categorical variables, and Mann-Whitney U test was used to determine p values for continuous variables.

Supplementary references:

1. Rinchai D, Roelands J, Toufiq M, Hendrickx W, Altman MC, Bedognetti D, et al. BloodGen3Module: Blood transcriptional module repertoire analysis and visualization using R. Bioinformatics. 2021.

2. Heaton T, Rowe J, Turner S, Aalberse RC, de Klerk N, Suriyaarachchi D, et al. An immunoepidemiological approach to asthma: identification of in-vitro T-cell response patterns associated with different wheezing phenotypes in children. Lancet. 2005;365(9454):142-9.
3. Kusel MM, Holt PG, de Klerk N, Sly PD. Support for 2 variants of eczema. J Allergy Clin Immunol. 2005;116(5):1067-72.
4. Kusel MM, de Klerk NH, Holt PG, Kebadze T, Johnston SL, Sly PD. Role of respiratory viruses in acute upper and lower respiratory tract illness in the first year of life: a birth cohort study. Pediatr Infect Dis J. 2006;25(8):680-6.
5. Kusel MM, de Klerk NH, Kebadze T, Vohma V, Holt PG, Johnston SL, et al. Early-life respiratory viral infections, atopic sensitization, and risk of subsequent development of persistent asthma. J Allergy Clin Immunol. 2007;119(5):1105-10.
6. Holt PG, Rowe J, Kusel M, Parsons F, Hollams EM, Bosco A, et al. Toward improved prediction of risk for atopy and asthma among preschoolers: a prospective cohort study. J Allergy Clin Immunol. 2010;125(3):653-9, 9 e1-9 e7.
7. Kusel MM, Kebadze T, Johnston SL, Holt PG, Sly PD. Febrile respiratory illnesses in infancy and atopy are risk factors for persistent asthma and wheeze. Eur Respir J. 2012;39(4):876-82.
8. Teo SM, Mok D, Pham K, Kusel M, Serralha M, Troy N, et al. The infant nasopharyngeal microbiome impacts severity of lower respiratory infection and risk of asthma development. Cell Host Microbe. 2015;17(5):704-15.
9. Holt PG, Mok D, Panda D, Renn L, Fabozzi G, deKlerk NH, et al. Developmental regulation of type 1 and type 3 interferon production and risk for infant infections and asthma development. J Allergy Clin Immunol. 2019;143(3):1176-82 e5.
10. Pertea M, Kim D, Pertea GM, Leek JT, Salzberg SL. Transcript-level expression analysis of RNA-seq experiments with HISAT, StringTie and Ballgown. Nat Protoc. 2016;11(9):1650-67.
11. Ewels P, Magnusson M, Lundin S, Kaller M. MultiQC: summarize analysis results for multiple tools and samples in a single report. Bioinformatics. 2016;32(19):3047-8.
12. Lassmann T, Hayashizaki Y, Daub CO. SAMStat: monitoring biases in next generation sequencing data. Bioinformatics. 2011;27(1):130-1.
13. Klavus A, Kokla M, Noerman S, Koistinen VM, Tuomainen M, Zarei I, et al. "notame": Workflow for Non-Targeted LC-MS Metabolic Profiling. Metabolites. 2020;10(4).
14. Kirwan JA, Broadhurst DI, Davidson RL, Viant MR. Characterising and correcting batch variation in an automated direct infusion mass spectrometry (DIMS) metabolomics workflow. Anal Bioanal Chem. 2013;405(15):5147-57.
15. Broadhurst D, Goodacre R, Reinke SN, Kuligowski J, Wilson ID, Lewis MR, et al. Guidelines and considerations for the use of system suitability and quality control samples in mass spectrometry assays applied in untargeted clinical metabolomic studies. Metabolomics. 2018;14(6):72.
16. Westerhuis JA, van Velzen EJ, Hoefsloot HC, Smilde AK. Multivariate paired data analysis: multilevel PLSDA versus OPLSDA. Metabolomics. 2010;6(1):119-28.
17. Liquet B, Le Cao KA, Hocini H, Thiebaut R. A novel approach for biomarker selection and the integration of repeated measures experiments from two assays. BMC Bioinformatics. 2012;13:325.
18. Rohart F, Gautier B, Singh A, Le Cao KA. mixOmics: An R package for 'omics feature selection and multiple data integration. PLoS Comput Biol. 2017;13(11):e1005752.
19. Lê S, Josse J, Husson F. FactoMineR: An R Package for Multivariate Analysis. 2008. 2008;25(1):18.
20. Robinson MD, McCarthy DJ, Smyth GK. edgeR: a Bioconductor package for differential expression analysis of digital gene expression data. Bioinformatics. 2010;26(1):139-40.
21. Robinson MD, Oshlack A. A scaling normalization method for differential expression analysis of RNA-seq data. Genome Biol. 2010;11(3):R25.
22. Risso D, Ngai J, Speed TP, Dudoit S. Normalization of RNA-seq data using factor analysis of control genes or samples. Nat Biotechnol. 2014;32(9):896-902.
23. Love MI, Huber W, Anders S. Moderated estimation of fold change and dispersion for RNA-seq data with DESeq2. Genome Biol. 2014;15(12):550.
24. Ritchie ME, Phipson B, Wu D, Hu Y, Law CW, Shi W, et al. limma powers differential expression analyses for RNA-sequencing and microarray studies. Nucleic Acids Res. 2015;43(7):e47.

25. Zhang B, Horvath S. A general framework for weighted gene co-expression network analysis. Stat Appl Genet Mol Biol. 2005;4:Article17.
26. Langfelder P, Horvath S. WGCNA: an R package for weighted correlation network analysis. BMC Bioinformatics. 2008;9:559.
27. Yu G, He QY. ReactomePA: an R/Bioconductor package for reactome pathway analysis and visualization. Mol Biosyst. 2016;12(2):477-9.
28. Yu G, Wang LG, Han Y, He QY. clusterProfiler: an R package for comparing biological themes among gene clusters. OMICS. 2012;16(5):284-7.
29. Breuer K, Foroushani AK, Laird MR, Chen C, Sribnaia A, Lo R, et al. InnateDB: systems biology of innate immunity and beyond--recent updates and continuing curation. Nucleic Acids Res. 2013;41(Database issue):D1228-33.
30. Altman MC, Rinchai D, Baldwin N, Toufiq M, Whalen E, Garand M, et al. Development of a fixed module repertoire for the analysis and interpretation of blood transcriptome data. Nat Commun. 2021;12(1):4385.
31. Bacher R, Chu LF, Leng N, Gasch AP, Thomson JA, Stewart RM, et al. SCnorm: robust normalization of single-cell RNA-seq data. Nat Methods. 2017;14(6):584-6.
32. Margolin AA, Nemenman I, Basso K, Wiggins C, Stolovitzky G, Dalla Favera R, et al. ARACNE: an algorithm for the reconstruction of gene regulatory networks in a mammalian cellular context. BMC Bioinformatics. 2006;7 Suppl 1:S7.
33. Lachmann A, Giorgi FM, Lopez G, Califano A. ARACNe-AP: gene network reverse engineering through adaptive partitioning inference of mutual information. Bioinformatics. 2016;32(14):2233-5.
34. Alvarez MJ, Shen Y, Giorgi FM, Lachmann A, Ding BB, Ye BH, et al. Functional characterization of somatic mutations in cancer using network-based inference of protein activity. Nat Genet. 2016;48(8):838-47.
35. Aibar S, Gonzalez-Blas CB, Moerman T, Huynh-Thu VA, Imrichova H, Hulselmans G, et al. SCENIC: single-cell regulatory network inference and clustering. Nat Methods. 2017;14(11):1083-6.
36. Lambert SA, Jolma A, Campitelli LF, Das PK, Yin Y, Albu M, et al. The Human Transcription Factors. Cell. 2018;172(4):650-65.
37. Newman AM, Steen CB, Liu CL, Gentles AJ, Chaudhuri AA, Scherer F, et al. Determining cell type abundance and expression from bulk tissues with digital cytometry. Nat Biotechnol. 2019;37(7):773-82.
38. Regev A, Teichmann SA, Lander ES, Amit I, Benoist C, Birney E, et al. The Human Cell Atlas. Elife. 2017;6.
39. Stuart T, Butler A, Hoffman P, Hafemeister C, Papalexi E, Mauck WM, 3rd, et al. Comprehensive Integration of Single-Cell Data. Cell. 2019;177(7):1888-902 e21.
40. Strobl C, Boulesteix AL, Kneib T, Augustin T, Zeileis A. Conditional variable importance for random forests. BMC Bioinformatics. 2008;9:307.
41. Tolosi L, Lengauer T. Classification with correlated features: unreliability of feature ranking and solutions. Bioinformatics. 2011;27(14):1986-94.
42. Singh A, Shannon CP, Gautier B, Rohart F, Vacher M, Tebbutt SJ, et al. DIABLO: an integrative approach for identifying key molecular drivers from multi-omics assays. Bioinformatics. 2019;35(17):3055-62.
